# First Infusion Reactions are Mediated by FcγRIIIb and Neutrophils

**DOI:** 10.1007/s11095-018-2448-8

**Published:** 2018-06-27

**Authors:** Felix Weber, Daniel Breustedt, Sonja Schlicht, Claas A. Meyer, Jens Niewoehner, Martin Ebeling, Per-Ola Freskgard, Peter Bruenker, Thomas Singer, Michael Reth, Antonio Iglesias

**Affiliations:** 1 Roche Pharmaceutical Research and Early Development, Pharmaceutical Sciences, Roche Innovation Center Basel, F. Hoffmann-La Roche Ltd., Bldg 93 Room 5.10, Grenzacherstrasse 124, 4070 Basel, CH Switzerland; 2 Novartis Pharma AG, Novartis Institutes for Biomedical Research, Basel, Switzerland; 3 Small Molecule Research, Therapeutic Modalities, Roche Innovation Center Basel, Basel, Switzerland; 4 Large Molecule Research, Therapeutic Modalities, Roche Innovation Center Munich, Munich, Germany; 5 Neuroscience, Ophthalmology and Rare Diseases Discovery and Translational Area, Roche Innovation Center Basel, Basel, Switzerland; 6 Large Molecule Research, Roche Innovation Center Zurich, Schlieren, Switzerland; 7 Institute of Biology III (Molecular Immunology), University of Freiburg, Freiburg im Breisgau, Germany

**Keywords:** human FcγRIIIb, humanized mouse model, immunotoxicology, infusion reactions, neutrophils

## Abstract

**Purpose:**

Administration of therapeutic monoclonal antibodies (mAbs) is frequently accompanied by severe first infusion reactions (FIR). The mechanism driving FIR is still unclear. This study aimed to investigate the cellular and molecular mechanisms causing FIR in humanized mouse models and their potential for evaluating FIR risk in patients.

**Methods:**

Mice humanized for Fc gamma receptors (FcγRs) were generated by recombination-mediated genomic replacement. Body temperature, cytokine release and reactive oxygen species (ROS) were measured to assess FIR to mAbs.

**Results:**

Infusion of human mAb specific for mouse transferrin receptor (HamTfR) into FcγR-humanized mice, produced marked transient hypothermia accompanied by an increase in inflammatory cytokines KC and MIP-2, and ROS. FIR were dependent on administration route and Fc-triggered effector functions mediated by neutrophils. Human neutrophils also induced FIR in wild type mice infused with HamTfR. Specific knock-in mice demonstrated that human FcγRIIIb on neutrophils was both necessary and sufficient to cause FIR. FcγRIIIb-mediated FIR was abolished by depleting neutrophils or blocking FcγRIIIb with CD11b antibodies.

**Conclusions:**

Human FcγRIIIb and neutrophils are primarily responsible for triggering FIR. Clinical strategies to prevent FIR in patients should focus on this pathway and may include transient depletion of neutrophils or blocking FcγRIIIb with specific mAbs.

**Electronic supplementary material:**

The online version of this article (10.1007/s11095-018-2448-8) contains supplementary material, which is available to authorized users.

## Introduction

Monoclonal antibodies (mAbs) constitute an impressively effective class of biological drugs in the treatment of a number of severe conditions, including cancer, immune disorders and infections (1). However, the broad use of mAbs during the last decades has revealed associated risks, primarily related to infusion reactions (2–4). While most infusion reactions are mild to moderate (e.g., skin rash, nausea, chill), in some patients these can be severe or life-threatening, e.g., anaphylactoid reactions or cytokine storm (2–4). Infusion reactions normally occur in the hours after first or second infusions (5) and the incidence varies considerably from less than 5% of treated patients affected (Omalizumab, Natalizumab, Cetuximab) to more than 20% (Infliximab, Rituximab, Trantuzumab) (6,7).

Infusion reactions are normally a primary phenomenon, also known as first infusion reactions (FIR), and in most cases their incidence decreases significantly in subsequent infusions. Secondary infusion reactions (SIR) can result from accumulating anti-drug antibodies (ADAs) causing anaphylactoid reactions following repeated administrations of mAbs. SIRs resemble acute systemic anaphylaxis as mediated by immunoglobulin G (IgG) and are triggered mainly by neutrophils, but can also be induced by monocyte/macrophages in mice (8). In contrast, in the absence of preexisting ADAs the pathogenic factors contributing to FIR are largely unknown. Given the strong negative impact of FIR on the successful development of therapeutic mAbs, understanding the underlying mechanisms is of uppermost importance.

For most therapeutic mAbs, preclinical studies in rodents and primates, even if highly reflective of human pharmacodynamics, are poorly predictive of human infusion reactions and toxicology (9,10). The study of infusion reactions in mouse models is hampered by intrinsic differences between the human and mouse sets of Fc gamma receptors (FcγRs). Humans display FcγRIIa/c in monocytes/macrophages and granulocytes, FcγRIIIa in monocytes and natural killer (NK) cells, and glycosylphosphatidylinositol (GPI)-anchored FcγRIIIb exclusively in neutrophils (11). Mice express FcγRIII on monocytes/macrophages, NK cells and neutrophils, FcγRIV in monocyte/macrophages and neutrophils and they lack homologue receptors for human FcγRIIa/c and FcγRIIIb (11). In addition, human FcγRIIIb is not bound by mouse IgG (12) and the affinities of different IgG subclasses for their FcγRs are different in the two species (13). Human FcγRIIIb lacks intracellular sequences and is anchored in the cell membrane via a GPI tail. Thus, intracellular signaling through FcγRIIIb is not mediated by the FcRγ common chain but has to be aided by other associated proteins. Blocking experiments have shown that the adhesion molecule macrophage-1 antigen (Mac-1, CD11b) mediates FcγRIIIb signaling (14,15). Finally, polymorphic FcγR variants exist in humans, which have no counterparts in mouse FcγRs and result in strong differences in the affinity for IgG proteins (16).

Here we describe the implementation of an *in vivo* system apt to predict and assess risks associated with FIR. The system makes use of a humanized mouse model expressing the main four human activating low-affinity FcγRs: FcγRIIa, FcγRIIIa, FcγRIIc and FcγRIIIb. The rationale of this approach relies on the fact that most infusion reactions triggered by therapeutic mAbs involve their interaction with FcγRs leading to activation of macrophages, basophils, antibody-dependent cellular cytotoxicity (ADCC), cytokine release and anaphylaxis (11). Given the intrinsic differences between mice and humans in FcγR number, cellular distribution and affinity to IgG, mice humanized for FcγR molecules provide an adequate system to understand and predict *in vivo* the risk of therapeutic mAbs to elicit FIR.

## Materials and Methods

### Mice

C57BL/6 mice (8–12 weeks old) used as wild type control mice were purchased from Charles River (Lyon, France). Mice strains HFCGR2–3, where mouse genes *Fcgr3* and *Fcgr4* were replaced by their human counterparts *FCGR2A*, *FCGR2C*, *FCGR3A,* and *FCGR3B*, and HFCGR3B, where mouse *Fcgr4* was replaced by human *FCGR3B* were generated in house.

**Fig. 1:**
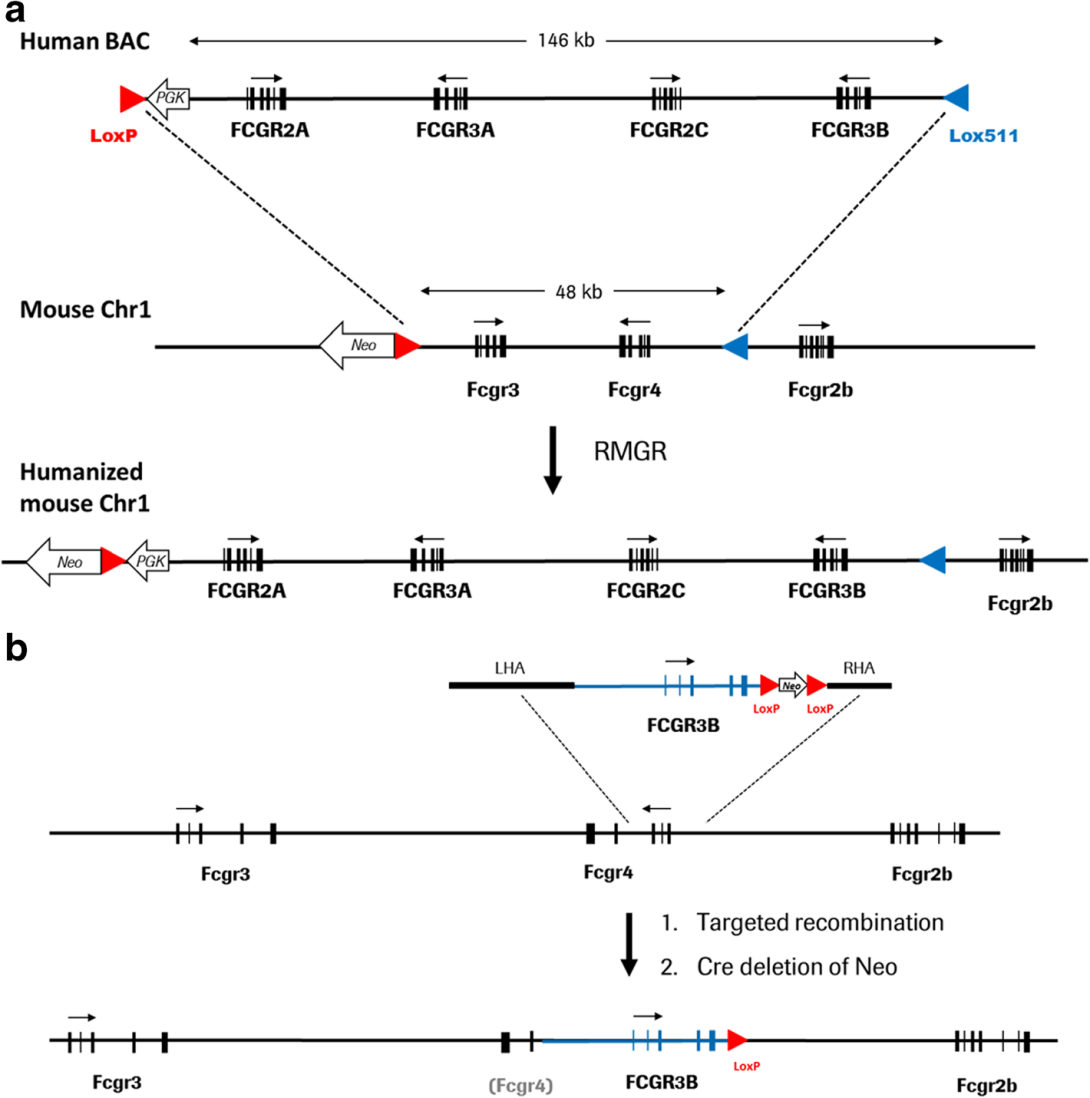
**Construction of humanized mouse strains HFCGR2–3 and HFCGR3B**. Representations not drawn to scale. (**a**), HFCGR2–3: Recombination-mediated genomic replacement (RMGR) of mouse genes *Fcgr3* and *Fcgr4* with human genes *FCGR2A*, *FCGR2C*, *FCGR3A* and *FCGR3B*. Upper line: BAC vector used encompassing human 146 kb sequence from 1:161494582 to 1:161640325 of human Chr1_q23.3, in GRCh38. Middle line: replaced 48 kb genomic region between positions 1:171015025 to 1:171062982 of mouse Chr1 in mm10 GRCh38. Lower line: humanized mouse locus with inserted human FcgR genes. (**b**), HFCGR3B: Targeted gene replacement of mouse *Fcgr4* gene with the human *FCGR3B* gene. Upper line: targeting vector composed of 13 kb sequence from 1:161623196 to 1:161636203 of human Chr1_q23.3, in GRCh38 bearing the human *FCGR3B* gene and flanking mouse sequences adjacent to the first and third Exon of mouse *Fcgr4* gene. Middle line: Scheme of the mouse *Fcgr* locus. Lower line: targeted mouse locus with human *FCGR3B* gene replacing the inactivated mouse *Fcgr4* gene (grey in parenthesis). Human genes are indicated in big capitals, mouse genes in small capitals. LHA and RHA indicate left homology and right homology arm, respectively. Red and blue arrowheads represent LoxP and Lox511 elements, respectively. White block arrows indicate Neo gene or PGK promoter. Small arrows indicate direction of transcription. The allelic variants of the human FcγR genes used here are *FCGR2B* (131R), *FCGR2C* (Stop variant), *FCGR3A* (128F) and *FCGR3B* (NA1).

FcγR-humanized HFCGR2–3 mice were generated by recombination-mediated genomic replacement (RMGR). They were produced by exchange of a 48 kb genomic region, encompassing mouse *Fcgr3* and *Fcgr4* gene, from murine chromosome 1 with a genomic DNA fragment of 146 kb from human chromosome 1 that contained human *FCGR2A*, *FCGR3A*, *FCGR2C* and *FCGR3B* genes (Fig. 1a). The HFCGR3B mutant mouse line was created by targeted gene replacement of mouse *Fcgr4* by the genomic version of human *FCGR3B* (Fig. 1b). The targeting strategy is described in detail in the Supplementary Materials and Methods and Fig. S1 and Fig. S2.

C57BL/6 mice served as wild type controls. All mice were bred and housed in our animal facility. Mice were maintained in a temperature controlled (22°C ± 2) facility, with a 12-h light/dark cycle and food and water supplied *ad libitum*. All animal procedures were performed in strict adherence to the Swiss federal regulations on animal protection and approved by the appropriate governmental authorities, to the rules of the AAALAC and with the explicit approval of the local veterinary authority (permission number 1902).

### First Infusion Reactions (FIR)

To study FIR upon primary administration of therapeutic antibodies, we used a human mAb specific for the murine transferrin receptor (TfR), an antigen widely present in recipient mice. FIR were elicited by intravenous (i.v.) injection of the indicated dose of HamTfR mAb in 100 μL of buffer solution (20 mM Histidine, 140 mM NaCl, pH 6.0) in C57BL/6 wild type mice and HFCGR2–3 or HFCGR3B mutant mice.

Body temperature was measured using the IPTT300 telemetry system and the DAS7006S Handheld Reader and Wireless Communications Module (Biomedic Data Systems Inc). In general, groups of five mice per condition were investigated. After an observation period of 2 h mice were sacrificed and a tail blood sample was taken for serum cytokine analysis. Cytokine analyses were performed using Mouse Cytokine Antibody Array, Panel A (R&D Systems) according to the manufacturer’s instructions.

### Imaging

*In vivo* imaging of reactive oxygen species (ROS) was performed using a PerkinElmer IVIS SpectrumCT and PerkinElmer inflammation probe. Mice were anaesthetized for analysis and the PerkinElmer inflammation probe injected intraperitoneally in a volume of 170 μL per mouse. After 10 min incubation, mice were imaged with an exposure time of 5 min at F1. Image quantification was performed using PerkinElmer Living Image software.

### Neutrophil Depletion and CD11b Blocking

For depletion of neutrophils, a Ly6G^+^ cell depleting antibody (clone NIMP-R14, kindly provided by Stefan Martin, Freiburg University Medical Center) was used. 125 μg of antibody were injected intraperitoneally 24 h prior to the experiment. The efficacy of neutrophil depletion in peripheral blood was confirmed by flow cytometry with a BD LSRFortessa™ Cell Analyzer (BD Biosciences) data analysis with FlowJo software (TreeStar). For blocking of CD11b, we used a blocking antibody (clone M1/70, Biolegend) (17). 100 μg antibody was injected 24 h prior to the FIR experiments.

### Statistical Analysis

Data were analyzed using GraphPad Prism (GraphPad Software Inc.). Temperature telemetry data are represented as mean ± SEM. Cytokine data depict the percentage of the assay internal (technical) positive control after subtraction of the relevant control group. Statistical significance was defined at a *P* value <0.05.

## Results

### Characterization of HFCGR2–3 and HFCGR3B Mouse Lines

The construction of humanized mouse lines HFCGR2–3 and HFCGR3B is depicted schematically in Fig. 1 and described in detail in Fig. S1 and Fig. S2. Both HFCGR2–3 and HFCGR3B mice were viable, fertile, free of inflammatory symptoms in the absence of challenge and stable for more than 10 generations. HFCGR2–3 mice display an expression pattern of human FcγRs resembling the human expression and cellular distribution pattern. HFCGR2–3 mice express human CD16 in blood monocytes, NK cells as well as neutrophils and human CD32 in blood monocytes and neutrophils, while lacking expression of mouse FcγRIII and FcγRIV (Fig. S3, Fig. S4B and S4C). HFCGR3B mice express human FcγRIIIb only in neutrophils and lack expression of mouse FcγRIV (Fig. S4).

### Characterization of FIR in FcγR-Humanized Mice

**Fig. 2:**
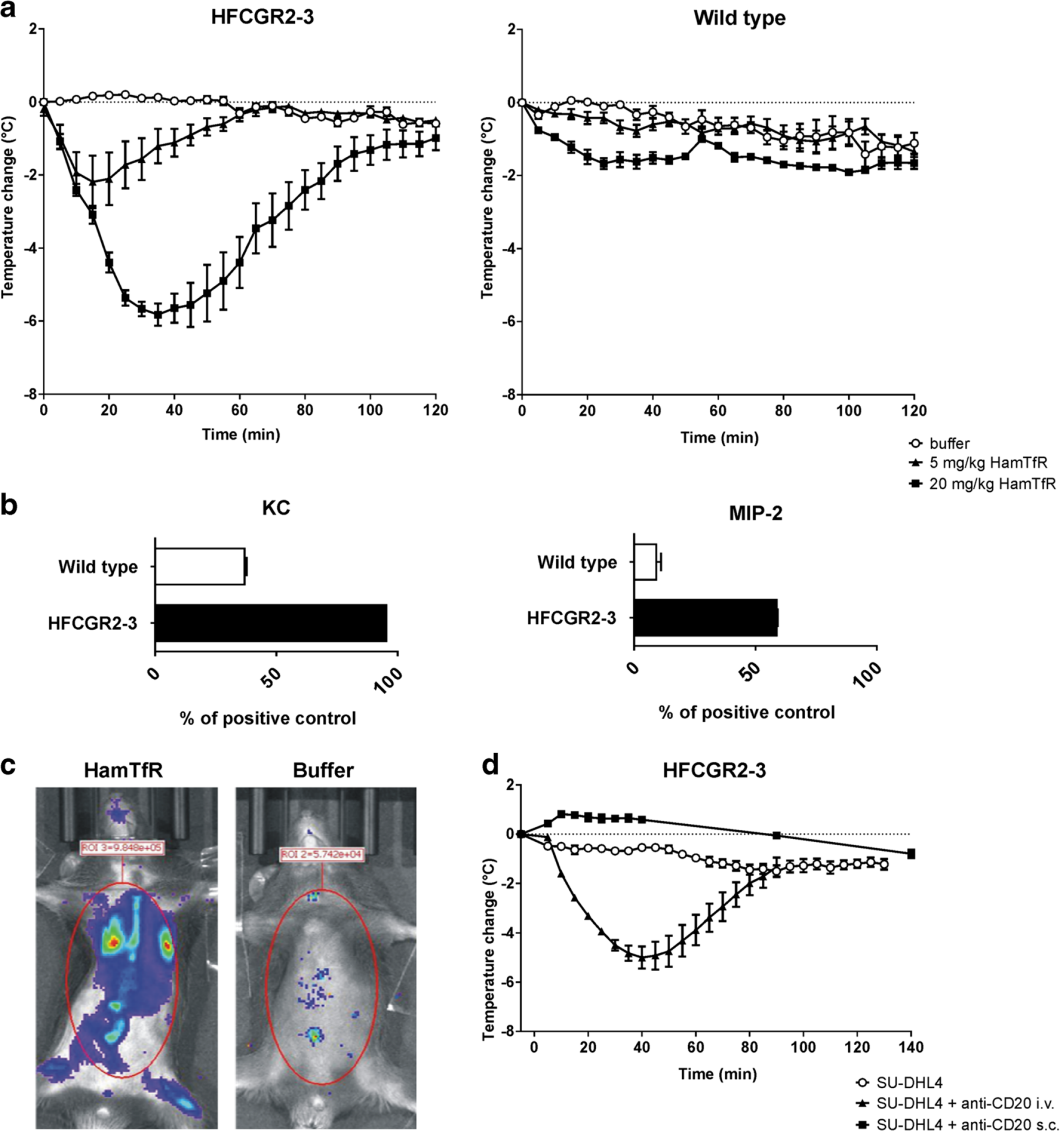
**Humanized HFCGR2–3 mice display FIR upon mAb injection**. (**a**), Change of body temperature of HFCGR2–3 (left panel) and wild type (right panel) mice injected with either buffer (open circles), 5 mg/kg (black triangles) or 20 mg/kg (black squares) of HamTfR antibody over a period of 2 h. (**b**), Serum levels of inflammatory cytokines KC (left panel) and MIP-2 (right panel) 2 h after infusion of 20 mg/kg HamTfR antibody in HFCGR2–3 and wild type mice. Serum levels are expressed as percentage of the assay’s internal positive control as described in Material and Methods and in Fig. S7. (**c**), In vivo production of ROS in HFCGR2–3 mice upon infusion of 20 mg/kg HamTfR antibody (left panel) or solvent buffer (right panel). (**d**), FIR as caused by infusing a mAb with different target specificity. Change of body temperature of HFCGR2–3 mice 2 h after injection of CD20-expressing human lymphoma cell line SU-DHL4 (black squares) or SU-DHL4 cells pre-incubated with human anti-CD20 antibody Rituximab injected i.v. (black triangles) or SU-DHL4 cells pre-incubated with Rituximab injected s.c. (open circles).

In the absence of previous priming, i.v. infusion of HamTfR caused a mild temperature decrease in C57BL/6 wild type mice [wild type buffer, −0.100 (0.04) *vs*. wild type HamTfR, −1.675 (0.15); mean difference (MD), 1.575 (0.17); 95% confidence interval (CI), 1.25–1.89; *t*_22_ = 10.11, *p* < 0.0001] followed by complete recovery after 2 h (Fig. 2a). In contrast, HFCGR2–3 mice displayed stronger symptoms characterized by a dose-dependent, drastic temperature drop [HFCGR2–3 buffer, −0.133 (0.09) *vs*. HFCGR2–3 HamTfR, −5.822 (0.30); MD, 5.96 (0.28); CI, 5.37–6.54; *t*_19_ = 21.30, *p* < 0.0001] and up-regulation of inflammatory cytokines KC and MIP-2 (Fig. 2a and b). Scrutiny of 40 mouse cytokines revealed KC and MIP-2 as the two cytokines consistently displaying a significant concentration increment (see Supplementary Materials and Methods and Table SIII). Additionally, we performed *in vivo* analyses of induction of ROS as a common marker of innate immune cell activation. Injection of HamTfR mAb into HFCGR2–3 mice resulted in a strong induction of ROS production compared with the buffer control (Fig. 2c).

The observed FIR-like symptoms developed following i.v. administration of HamTfR, whereas subcutaneous (s.c.) injection caused no symptoms in HFCGR2–3 mice (Fig. S5A). I.v. infusion of larger amounts of human mAbs (up to 250 mg/kg) that do not bind any recipient target, such as Synagis (anti Respiratory Syncytial Virus) or Xolair (anti-human IgE), caused no FIR in HFCGR2–3 mice (Fig. S5B). Finally, no signs of FIR were observed when a PGLALA variant of HamTfR lacking FcγR binding was used (Fig. S5C).

To test the potential of infused human mAbs binding a different cellular target to induce FIR in HFCGR2–3-humanized mice, we used anti-CD20 human mAbs. I.v. application of anti-CD20 mAb Rituximab in human CD20-expressing transgenic mice did not cause FIR (Fig. S6A). The absence of FIR with this mAb is possibly related to the low levels of surface expression of transgenic human CD20 in these mice. As shown in Fig. S6B and S6C*,* the density of cell surface human CD20 in B cells of transgenic mice is ten times lower than found in human B cells and hundred times lower than observed in human lymphoma cells, the actual target of these therapeutic mAbs. Therefore, we infused wild type mice with 1 × 10^7^ human SU-DHL-4 lymphoma cells that had been pre-incubated with Rituximab prior to injection. The i.v. transfer of SU-DHL-4 lymphoma cells pre-coated with Rituximab into HFCGR2–3 mice resulted in a temperature drop comparable to that caused by the infusion of HamTfR mAbs. The same amount of human lymphoma cells coated with Rituximab did not cause FIR when administered s.c. (Fig. 2d). This result confirmed our previous findings with s.c. application of HamTfR in HFCGR2–3 mice (Fig. S5A).

### Role of Neutrophils in Inducing FIR

**Fig. 3:**
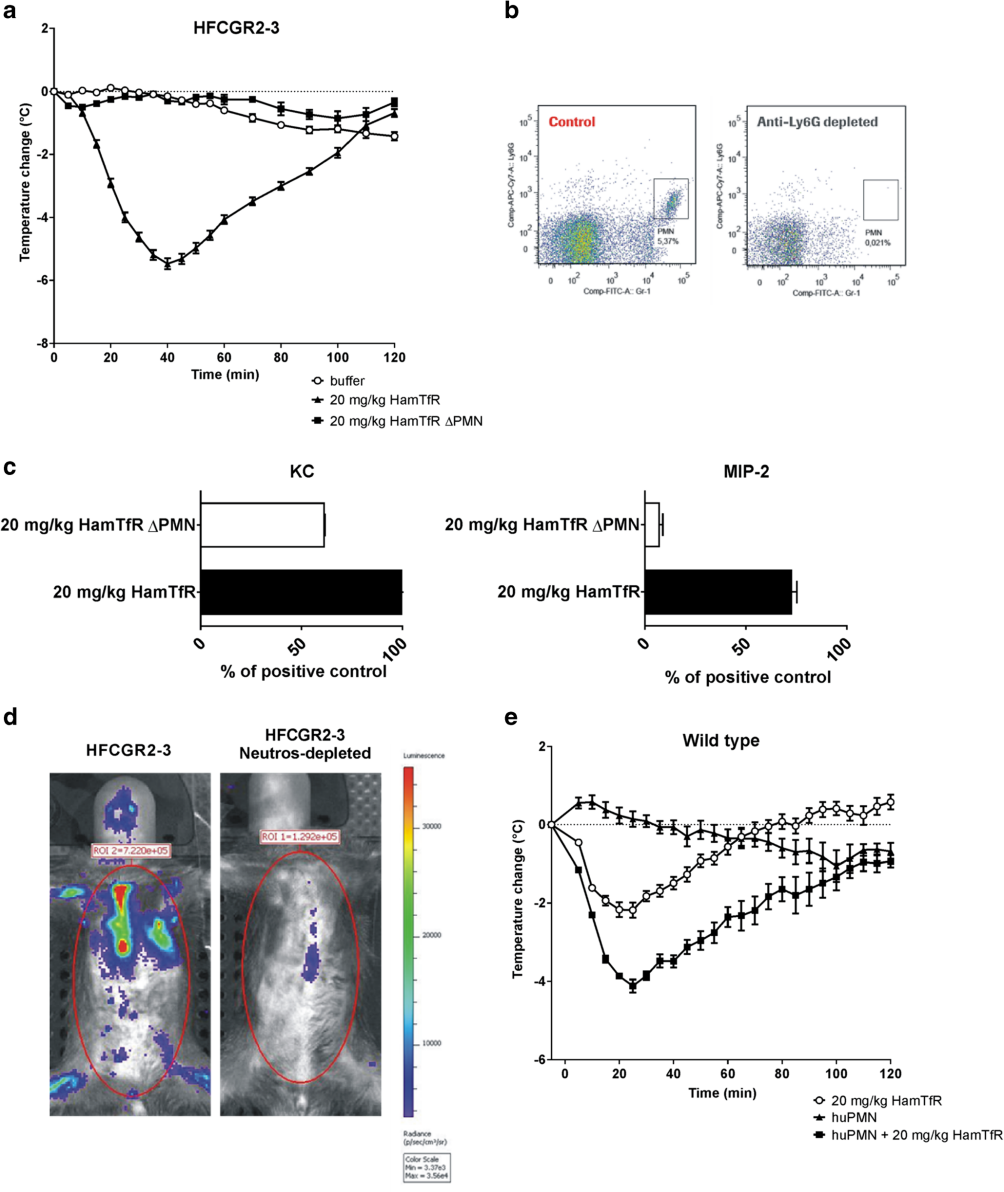
**Neutrophils are crucial for FIR in HFCGR2–3 mice** (**a**), Change of body temperature upon injection of 20 mg/kg HamTfR into HFCGR2–3 mice depleted of neutrophils (filled squares, ΔPMN) or left un-depleted (filled triangles). Un-depleted HFCGR2–3 mice injected with buffer instead of HamTfR served as control (open circles). (**b**), Depletion of neutrophils using mAb NIMP-R14 was confirmed by FACS analysis of peripheral blood of treated and control mice using FITC-labeled Gr-1 and APC-Cy7A labeled Ly6G mAbs. The neutrophil population is indicated by the square. (**c**), Serum levels of KC and MIP-2 of HFCGR2–3 mice depleted of neutrophils (ΔPMN) or left un-depleted after challenge with 20 mg/kg HamTfR. (**d**), Induction of ROS upon infusion of 20 mg/kg HamTfR mAb in HFCGR2–3 mice depleted (right panel) or non-depleted (left panel) of neutrophils (**e**), Change of body temperature of wild type mice adoptively transferred with purified human neutrophils and challenged with 20 mg/kg HamTfR (filled squares) or buffer control (filled triangles). Non-transferred mice challenged with 20 mg/kg HamTfR served as control (open circles).

To investigate the role of neutrophils in our FIR model HamTfR was infused (i.v.) into HFCGR2–3 mice depleted of neutrophils. In the absence of neutrophils, infusion of HamTfR, mAb failed to induce the typical FIR-related temperature drop, increased concentration of KC and MIP-2 and triggering of ROS production (Fig. 3a–d). To further substantiate the role of neutrophils in inducing FIR, purified human neutrophils were transferred into wild type mice followed by i.v. infusion of HamTfR mAb. As shown in Fig. 3e, this was sufficient to provoke a temperature drop stronger than in wild type mice treated with HamTfR alone and similar to that observed in HFCGR2–3-humanized mice.

### FcγRIIIb is Sufficient to Induce FIR

**Fig. 4:**
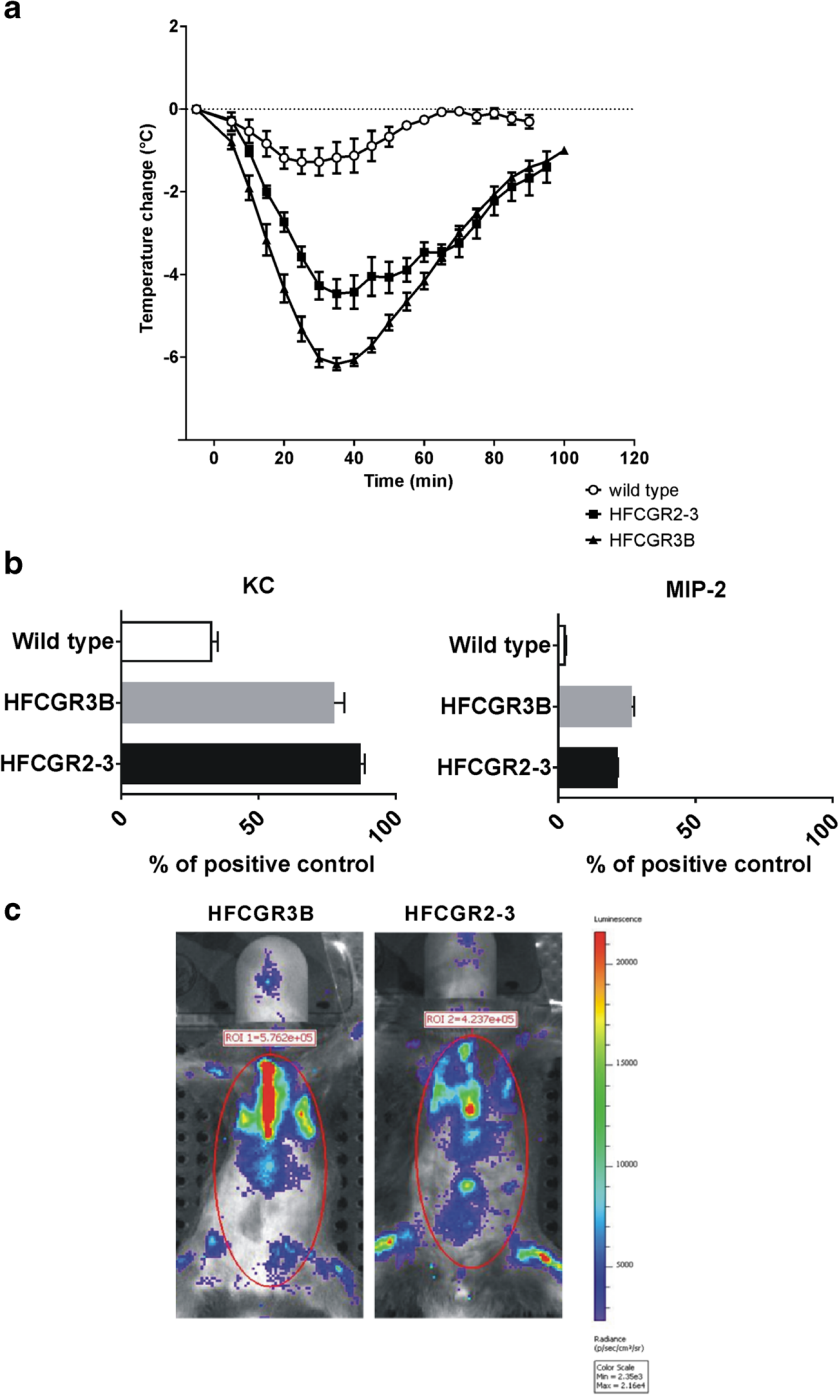
**HFCGR3B and HFCGR2–3 mice are similar in terms of experimental FIR.** (**a**), Change of body temperature of HFCGR2–3, HFCGR3B and wild type mice upon injection of 20 mg/kg HamTfR mAb. (**b**), Serum levels of KC and MIP-2 in HFCGR2–3, HFCGR3B and wild type mice treated with 20 mg/kg HamTfR mAb. (**c**), Induction of ROS in HFCGR2–3 and HFCGR3B mice upon infusion of HamTfR mAb.

Similar to humans, the Gr-1 positive neutrophils of HFCGR2–3-humanized mice express FcγRIIa (CD32) and FcγRIIIb (CD16b) (Fig. S3B–C). This expression thus accounts for the induction of FIR by neutrophils, as demonstrated by the experiments shown in Fig. 3. In contrast, expression of FcγRIII and FcγRIV, the murine homologues of human FcγRIIa and FcγRIIIa respectively, in mouse neutrophils is not sufficient to cause full blown FIR in wild type mice. Given that FcγRIIIb is found exclusively in neutrophils, we tested the relevance of this receptor for FIR by injecting HamTfR i.v. into FCGR3B mice. HamTfR provoked a rapid and strong hypothermia (Fig. 4a) and enhanced production of KC and MIP-2 (Fig. 4b) comparable to that caused in HFCGR2–3 mice. Likewise, *in vivo* imaging demonstrated that challenging HFCGR3B mice with HamTfR also caused rapid ROS production in a magnitude comparable to that seen in HFCGR2–3 mice (Fig. 4c). Thus we ascribe the induction of FIR to the triggering of neutrophils via binding of HamTfR to FcγRIIIb.

### Blocking of CD11b Prevents FIR

**Fig. 5:**
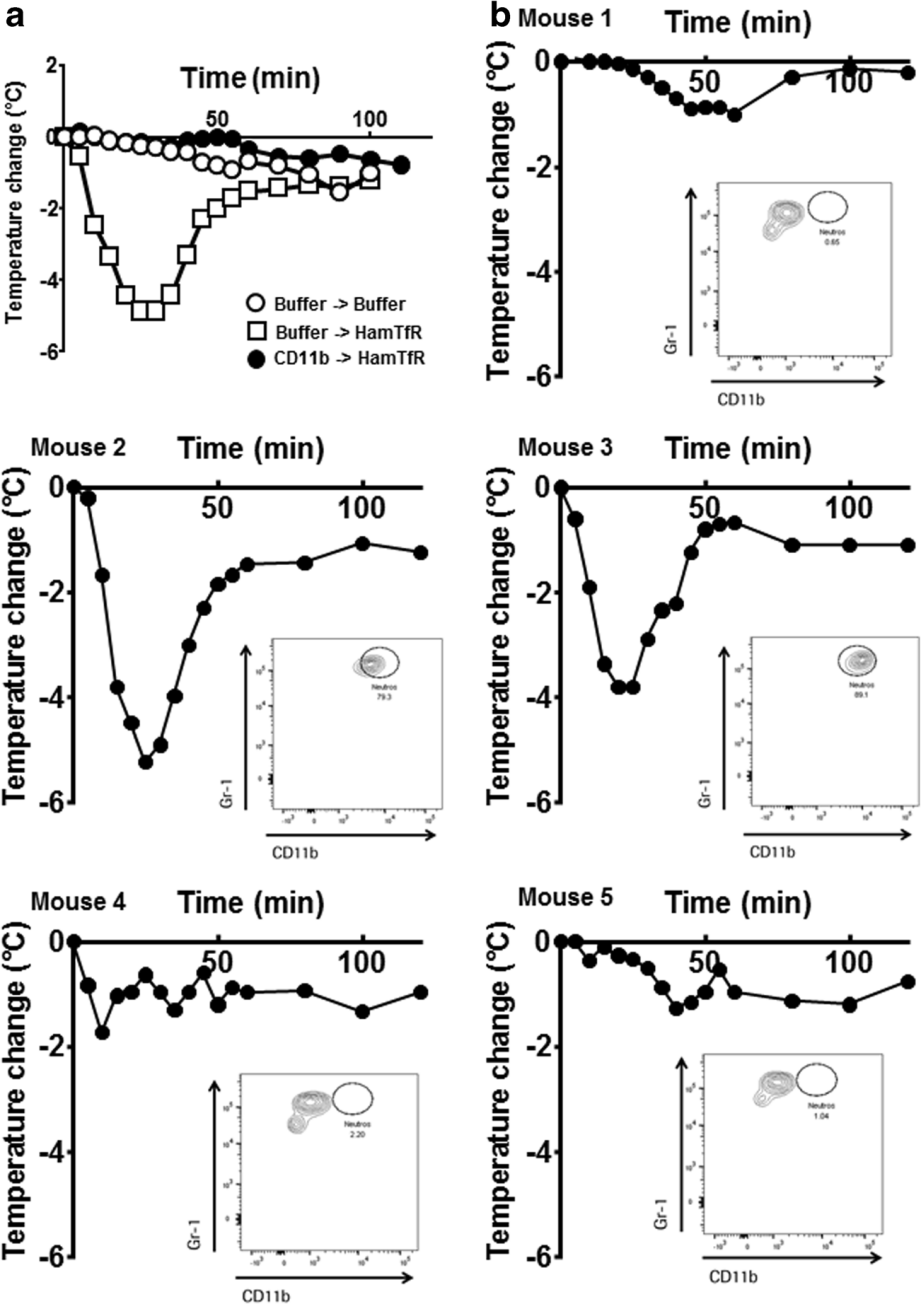
**Blocking of FcγR3b co-receptor CD11b can prevent FIR in HFCGR3B mice**. (**a**), Change of body temperature of HFCGR3B mice treated with CD11b blocking antibody M1/70 (filled circles) or buffer control (open circles and open squares) 24 h prior to injection of 20 mg/kg HamTfR (open squares and filled circles) or buffer control (open circles). Depicted are the means of five individual mice. (**b**), Change of body temperature of five individual HFCGR3B mice pre-treated with M1/70 and challenged with 20 mg/kg HamTfR mAbs. The inlays show FACS analyses of blood neutrophils after temperature measurement using Gr-1 antibodies to identify neutrophils in the gated population and PE-labeled M1/70 to detect unoccupied CD11b surface molecules.

To test the involvement of CD11b in our FIR model, HFCGR3B mice were treated with anti-CD11b mAb (M1/70) 24 h prior to treatment with HamTfR. Mice not pre-treated with anti-CD11b mAb displayed the typical rapid and transient drop in body temperature upon infusion of HamTfR (Fig. 5a). In mice pre-treated with anti-CD11b mAb two animals displayed a strong body temperature drop (Fig. 5b, mice 2 and 3), while three mice displayed only a mild temperature reduction typically observed in wild type animals (Fig. 5b, mice 1, 4 and 5). In FACS analyses of surface CD11b in neutrophils of the treated mice using PE-labelled M1/70 the bound unlabeled mAb will prevent PE-labelled M1/70 mAb from binding thus giving account on the degree of the blocking of CD11b by the unlabeled M1/70 mAb. This analysis revealed poor or totally absent occupancy with unlabeled M1/70 mAb in the two mice displaying full-blown temperature drop (mice 2 and 3 in Fig. 5b, inlays). In contrast, the density of M1/70-PE-stained neutrophils in the other three mice showing a mild temperature response was 10-fold lower (mice 1, 4 and 5in Fig. 5b), clearly indicating a strong occupancy of the CD11b surface molecule by the unlabeled M1/70 mAb.

## Discussion

Due to fundamental differences in the composition and cellular distribution of FcγRs in mice and humans, the study of clinical FIR, particularly in mouse models, can be difficult (11). The use of humanized mice expressing human activating low-affinity FcγRs has facilitated the study of their function *in vivo* and helped to determine their role in experimental disease models (11). Previous FcγRs-humanized mouse models combined different manipulated gene loci reproducing the full array of human FcγRs in mice lacking mouse FcγRs (18). However, more recently, Gillis and colleagues have developed a novel FcγR-humanized mouse strain using targeted genomic exchange, whereby mice deficient for the low-affinity mouse FcγR locus were generated and human FcγR were inserted into the equivalent locus (13). This model was used to study active and passive systemic anaphylaxis models and human FcγRIIa and neutrophils were identified as the main drivers of anaphylactic reactions (13). Here, we generated FcγR-humanized mouse models to investigate the interaction of the human low-affinity FcγRs with infused human IgG1 antibodies in FIR. Except for human FcγRIIb (not included in our models), the pattern of FcγR expression in our humanized mouse was similar to that described by Gillis *et al*. (13). Also, the potential role of FcγRIIb in mitigating FIR, as described for anaphylaxis (29), can be provided by mouse FcγRIIb in our model, given that human IgG1 has comparable affinity to human and mouse FcγRIIb (11). Whilst our findings confirm neutrophils as the main driver of FIR, we demonstrate that FcγRIIIb on neutrophils is the sole mediator of FIR. The finding that neutrophils were responsible for two distinct immune reactions driven by two different FcγRs indicated that our FcγR-humanized mouse models were capable of discriminating two well-defined and different immunological phenomena.

HFCGR2–3 mice infused with HamTfR (i.v.) experienced strong FIR-like symptoms, including rapid temperature drop, increased production of cytokines and ROS. IgG-induced anaphylaxis and anaphylactoid reactions are influenced by eosinophils, basophils and monocytes/macrophages but can also be triggered by neutrophils alone (8). KC and MIP-2 have been reported as the main neutrophil-recruiting cytokines elicited in acute inflammatory processes causing attraction of neutrophils to the injury site (19). Therefore, the inflammatory reactions reported here may reflect a murine correlate of human FIR. In the absence of antigenic priming, the induction of FIR in HFCGR2–3 mice but not in wild type mice suggests the involvement of human FcγRs. This was further substantiated by the absence of FIR when a PG-LALA mutant variant of the HamTfR lacking the capacity to interact with FcγR (20) was used. Furthermore, infused mAbs not binding to any resident target also failed to provoke FIR. Taken together, these findings indicate that the formation of an immune complex and its interaction with FcγRs are required for FIR. Additionally, FIR was dependent on administration route; only i.v administered mAbs caused a temperature drop and cytokine release. It is presently unknown whether this difference results from slower release into the circulation or from different processing of the immune complex in skin tissue *versus* blood. Nonetheless, given a comparable efficacy, the lack of FIR when therapeutic mAbs were given s.c. as compared to i.v. suggests the former as a safer administration route.

The TfR is distributed in various tissues and predominantly found in vascular endothelial cells, and as such it is expected to represent a target antigen easily accessible for i.v. infused (anti-TfR) antibodies. In contrast, surface antigen CD20 is restricted to B lymphocytes in the circulation and within immune organs. Surprisingly, infusion of anti-CD20 antibodies into human-CD20/HFCGR2–3 double-transgenic mice did not provoke FIR. However, the detected 10- and 100-fold lower cell surface density of human CD20 found in transgenic B cells compared with human B cells and human lymphoma cells suggest that target antigen crosslinking might influence neutrophil activation and FIR outcome. In fact, i.v. (but not s.c.) transfer of human lymphoma cells previously coated with Rituximab into HFCGR2–3 mice elicited a strong temperature drop. Therefore, we conclude that FIR unfolding is also a function of the cell surface density of target antigen bound by the infused antibodies.

With the infusion of HamTfR (or anti-human CD20 mAbs), abundant immune complexes are readily available to circulating leukocytes, especially neutrophils. Upon neutrophil activation, the high levels of ROS produced represent one of the most powerful effector functions. ROS are strong anti-microbial agents but at the same time may cause collateral damage in the host and can have a strong systemic pro-inflammatory effect (21–23). Upon neutrophil activation, intracellular granules are released and pro-inflammatory cytokines trigger a cascade leading to more cytokine release, thermic dysregulation, vasodilatation and release of neutrophils from the bone marrow (24). The exact mechanism by which released cytokines contribute to FIR remains to be clarified; however, KC and MIP-2 are likely to be involved. Neutrophils themselves are among the many cell types capable of producing KC and MIP-2 (25,26). These two cytokines promote neutrophil activation in an autocrine manner by activating integrins and general activation through CXCR2 (27). Along with ROS, which also mediates vasodilatation (28), secondary, anaphylactoid infusion reactions induced in mice primed with human antibodies, neutrophils have been shown to play a crucial role (8). Similarly, we demonstrated here that neutrophils are also the main cellular factors in FIR. Indeed, all FIR associated symptoms were abrogated when neutrophils were depleted in HFCGR2–3 mice prior to challenge with HamTfR. Conversely, human neutrophils transferred into wild type mice were also able to elicit FIR in combination with HamTfR.

Both murine FcγRIII and FcγRIV have been described to contribute to anaphylactoid reactions. Additionally, human FcγRIIa has been demonstrated to be the predominant FcγR involved in anaphylaxis (13,29). Since neutrophils in HFCGR2–3 mice expressed human FcγRIIa and FcγRIIIb it became necessary to discriminate experimentally between these two receptors in order to directly assess their contribution to FIR. Thus, we generated HFCGR3B knock-in mice, which expressed human FcγRIIIb as the only human FcγR in neutrophils and were fully susceptible to FIR. Indeed, in non-transgenic neutrophils the expression of mouse FcγRIII and FcγRIV was not sufficient to induce strong FIR, in spite of the fact that human IgG1 antibodies bind to murine FcγRIV with a 50-fold higher affinity (1 × 10^7^ M^−1^) (30) than to human FcγRIIIb (2 × 10^5^ M^−1^) (11). It has been shown that the rapid attachment of circulating human neutrophils to endothelial cells requires binding of immune complexes by FcγRIIIb (31). The cytokines KC and MIP2 are the murine homologues of human IL-8 and are known to specifically mediate neutrophil recruitment (19). Therefore, the observed specific increase in these two cytokines in our two FcγR-humanized mice supports the notion that recruitment of neutrophils is a central event during FIR. Thus, we report here for the first time a specific function of human FcγRIIIb on neutrophils as the central mediator of FIR upon infusion of mAbs with specificity for resident cellular target antigens.

Signal transduction of FcγRIIIb is mediated by accessory proteins such as CD11b/Mac-1 (19). Here we show that treatment of HFCGR3B mice with mAbs capable of blocking CD11b is sufficient to completely prevent the triggering of FIR. Thus, CD11b is involved in signaling via FcγRIIIb during neutrophil-mediated FIR. It is conceivable that CD11b-blocking antibodies may find application in the prophylaxis of acute FIR in patients. Taken into account that HFCGR3B mice express only human FcγRIIIb, a potential moderating effect of the other human FcγR competing for binding immune complexes could be missing in this system. But even if FIR in HFCGR3B represents a slight experimental exaggeration of real FIR in humans, the prophylactic strategy emerging from this work clearly points to depleting neutrophils or blocking FcγRIIIb signaling.

## Conclusion

The rapid drop in temperature, increase in KC and MIP-2 cytokines and production of ROS observed in our two FcγR-humanized mice suggest a rapid and systemic activation of neutrophils in response to the formation of immune complexes by mAb. This demonstrates for the first time that human FcγRIIIb in neutrophils is sufficient for triggering FIR. Furthermore, the results suggest that FIR as mediated by FcγRIIIb on neutrophils is triggered via signaling through the associated co-receptor CD11b. Taken together, the current investigation has helped to clarify some aspects of FIR caused by the primary infusion of therapeutic mAbs and may offer new clinical approaches to prevent potentially life-threatening effects of human antibody therapy.

## Electronic supplementary material

ESM 1 (PNG 286 kb)

High resolution image (TIF 699 kb)

ESM 2 (PNG 661 kb)

High resolution image (TIF 2047 kb)

ESM 3 (PNG 927 kb)

High resolution image (TIF 503 kb)

ESM 4 (PNG 638 kb)

High resolution image (TIF 378 kb)

ESM 5 (PNG 246 kb)

High resolution image (TIF 600 kb)

ESM 6 (PNG 272 kb)

High resolution image (TIF 835 kb)

ESM 7 (DOCX 67 kb)

## Abbreviations

ADA: Anti-drug antibodies
ADCC: Antibody-dependent cellular cytotoxicity
FcγR: Fc gamma receptor
FIR: First infusion reaction
GPI: glycosylphosphatidylinositol
HamTfR: Human anti-mouse transferrin receptor
IgG: Immunoglobulin G
i.v.: Intravenous
KC: Keratinocyte chemoattractant, CXCL1
mAbs: Monoclonal antibodies
MIP-2: Macrophage-inflammatory protein, CXCL2
NK cells: Natural killer cells
RMGR: Recombination-mediated gene replacement
ROS: Reactive oxygen species
s.c.: Subcutaneous
SIR: Second infusion reaction
TfR: Transferrin receptor
