# Supplementary figures and images for: First Infusion Reactions are Mediated by FcγRIIIb and Neutrophils
Source: Pharm Res. 2018 Jun 27;35(9):169. doi: 10.1007/s11095-018-2448-8 (PMC6021477; doi:10.1007/s11095-018-2448-8)

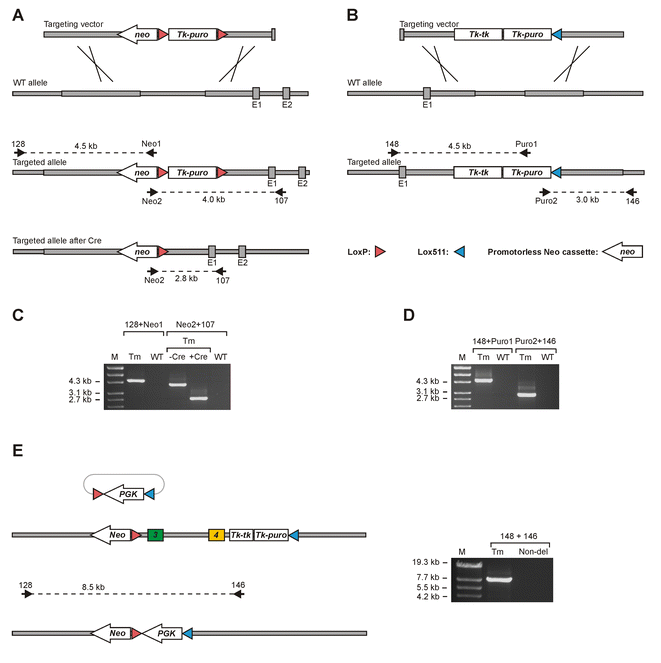

Supplement: Supplementary file 1 — (PNG 286 kb) [file 11095_2018_2448_Fig6_ESM.png]

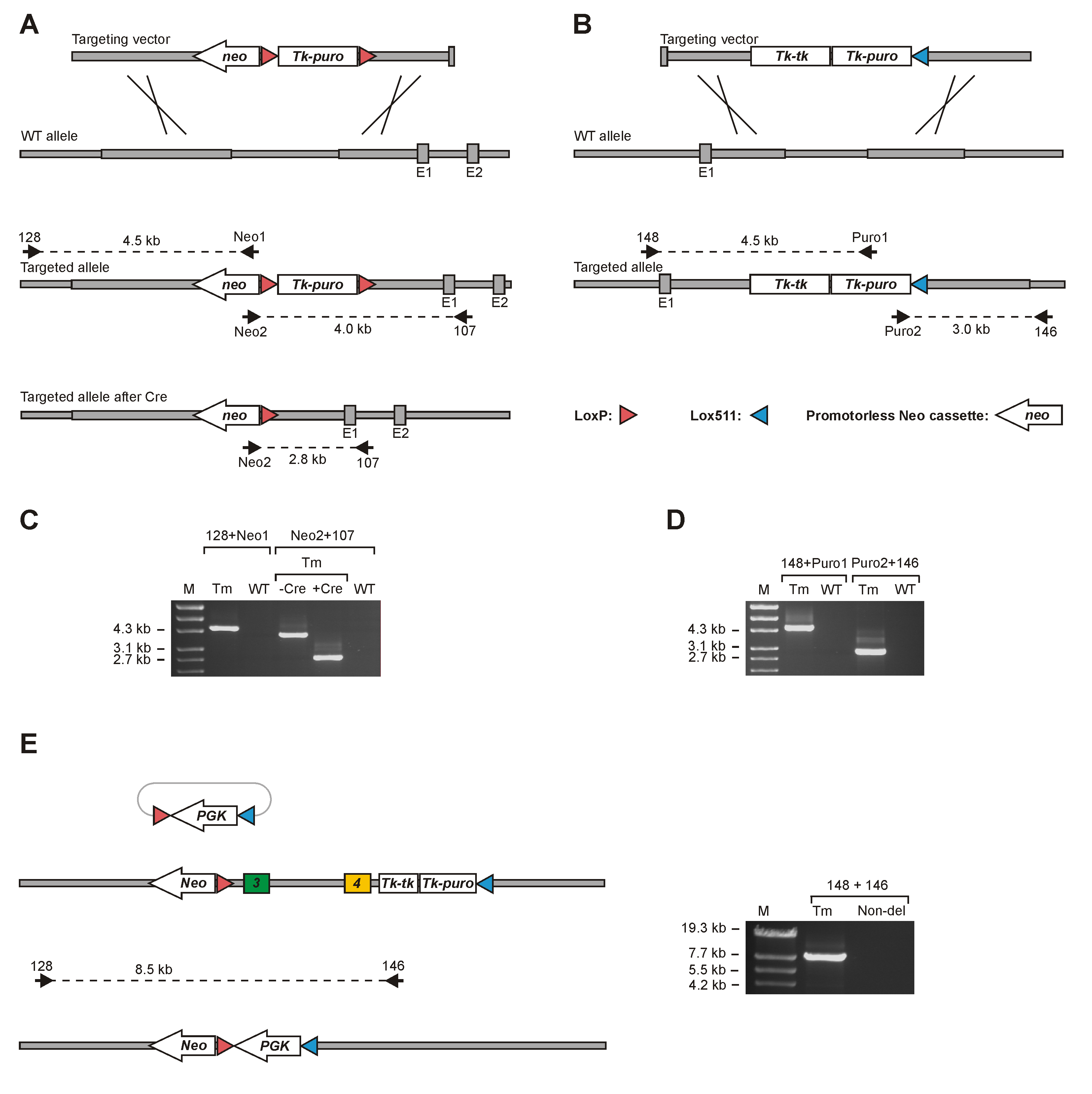

Supplement: Supplementary file 2 — High resolution image (TIF 699 kb) [file 11095_2018_2448_MOESM1_ESM.tif]

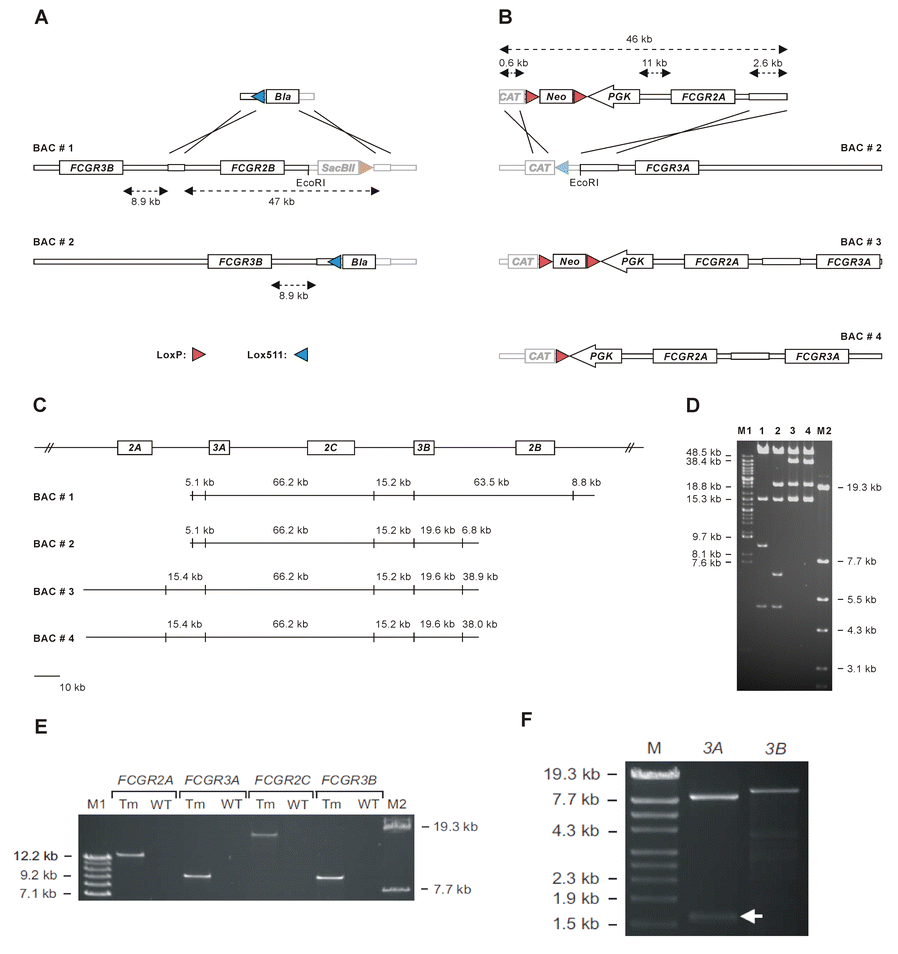

Supplement: Supplementary file 3 — (PNG 661 kb) [file 11095_2018_2448_Fig7_ESM.png]

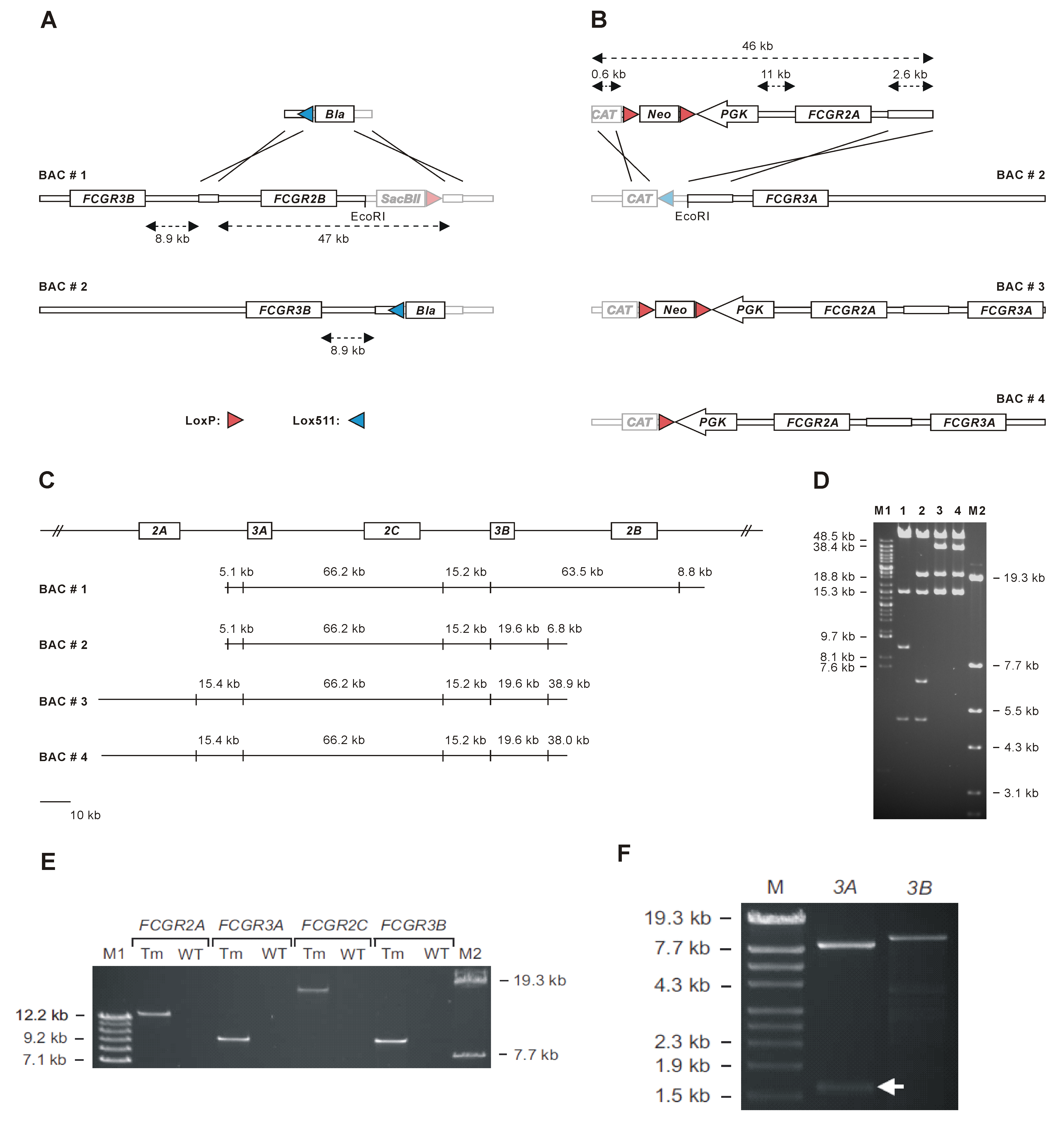

Supplement: Supplementary file 4 — High resolution image (TIF 2047 kb) [file 11095_2018_2448_MOESM2_ESM.tif]

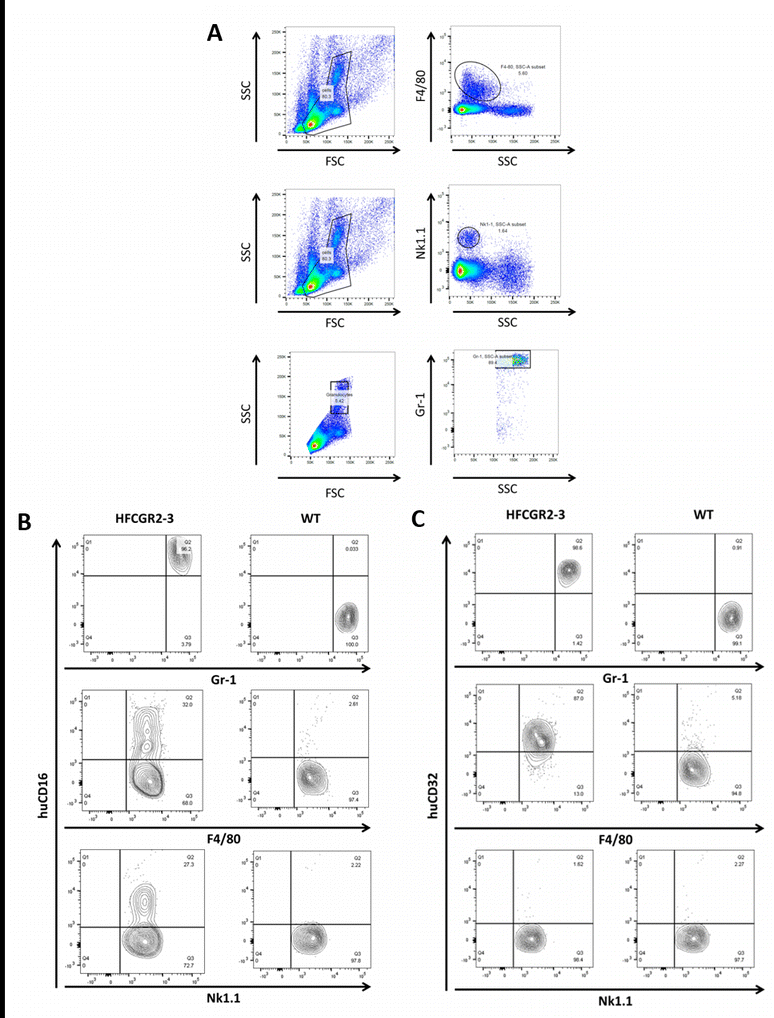

Supplement: Supplementary file 5 — (PNG 927 kb) [file 11095_2018_2448_Fig8_ESM.png]

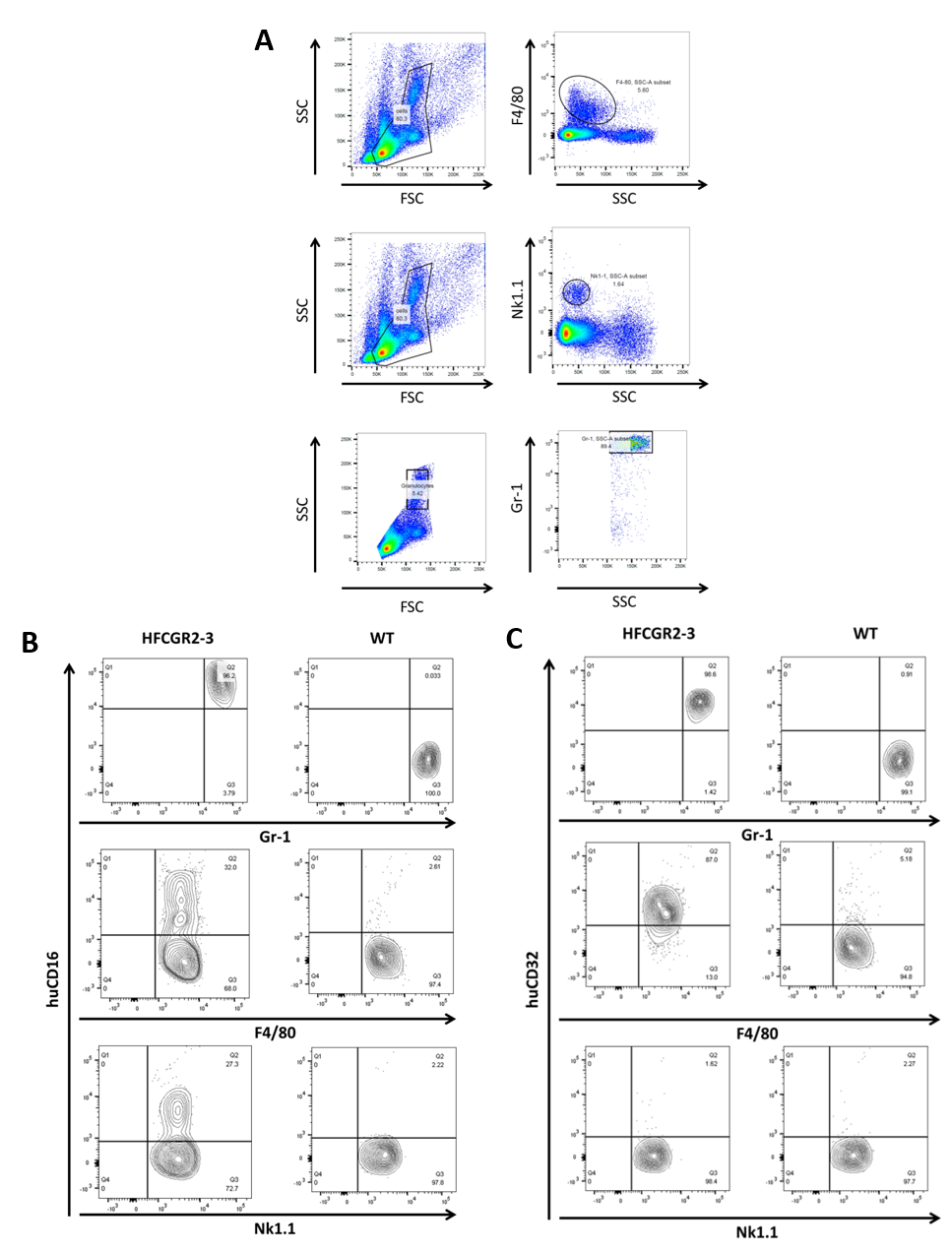

Supplement: Supplementary file 6 — High resolution image (TIF 503 kb) [file 11095_2018_2448_MOESM3_ESM.tif]

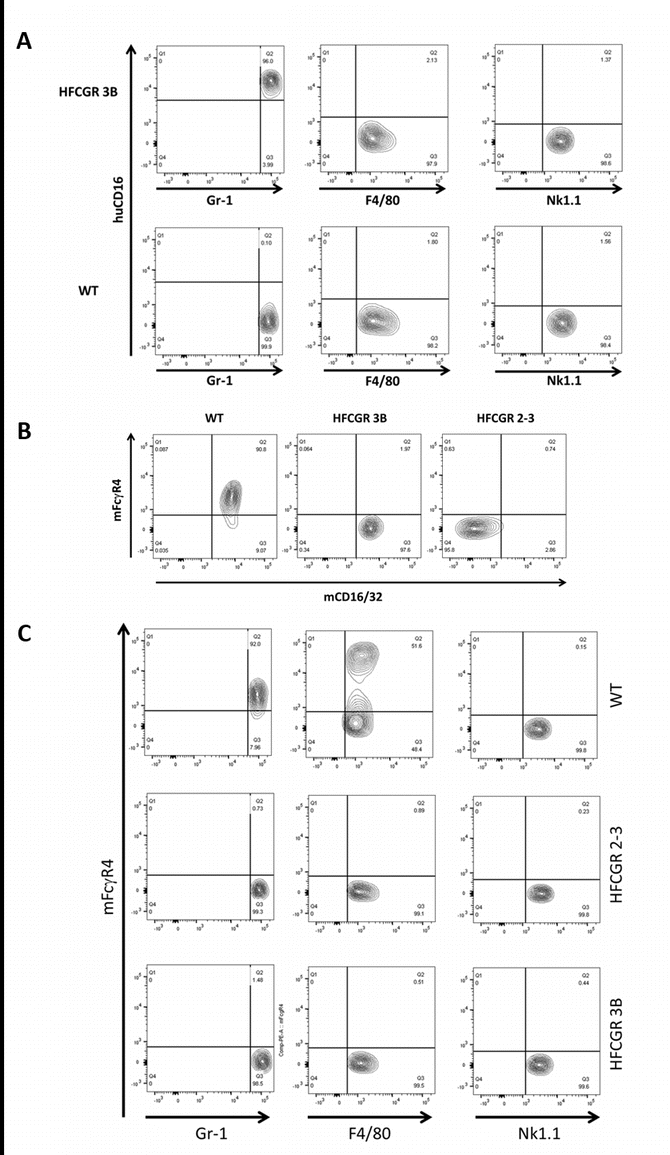

Supplement: Supplementary file 7 — (PNG 638 kb) [file 11095_2018_2448_Fig9_ESM.png]

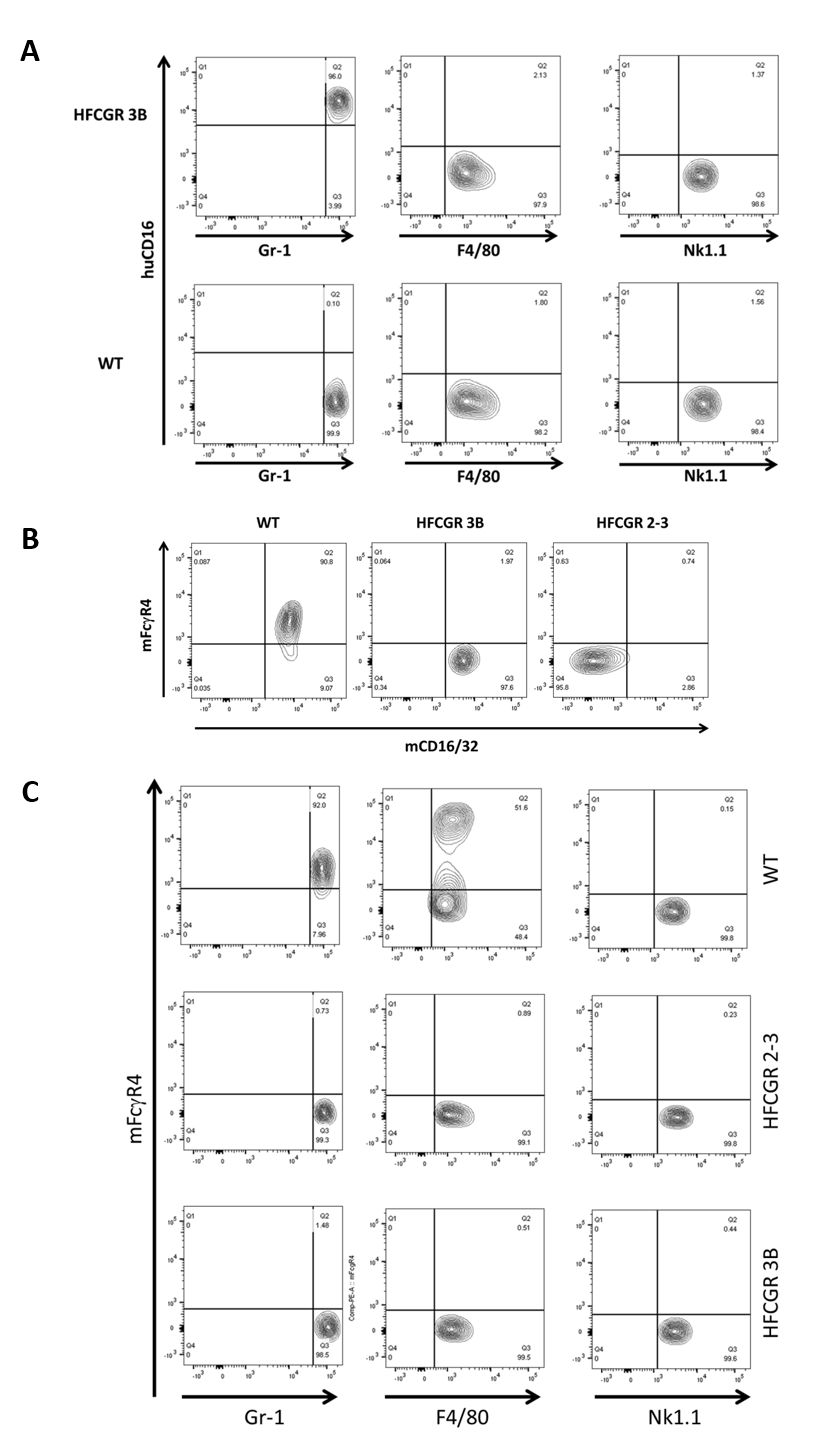

Supplement: Supplementary file 8 — High resolution image (TIF 378 kb) [file 11095_2018_2448_MOESM4_ESM.tif]

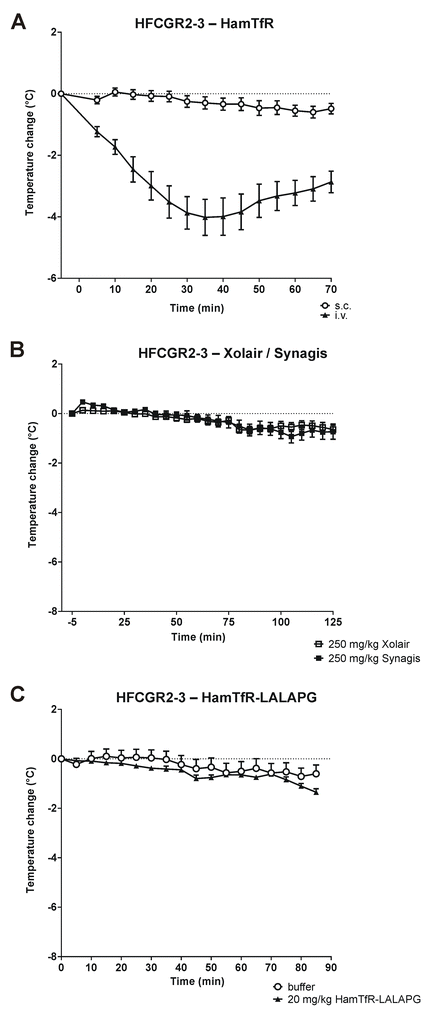

Supplement: Supplementary file 9 — (PNG 246 kb) [file 11095_2018_2448_Fig10_ESM.png]

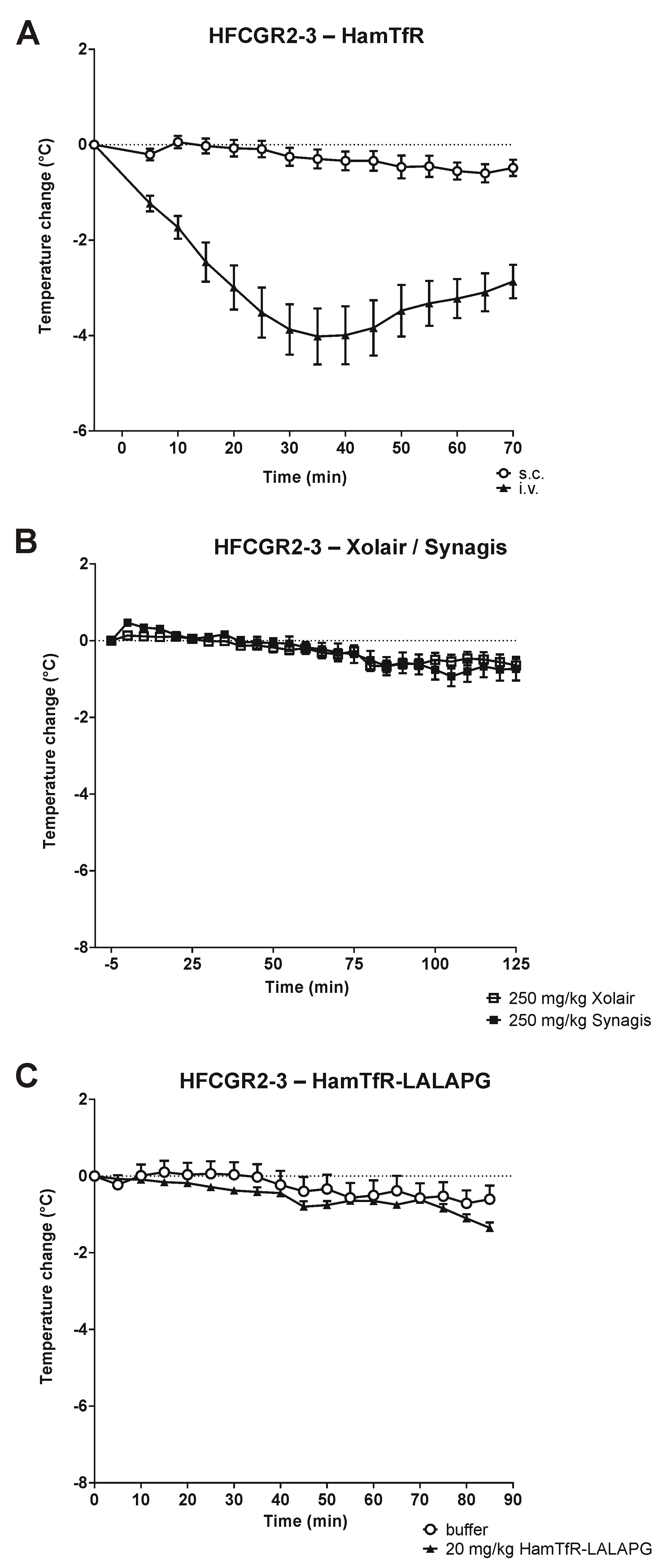

Supplement: Supplementary file 10 — High resolution image (TIF 600 kb) [file 11095_2018_2448_MOESM5_ESM.tif]

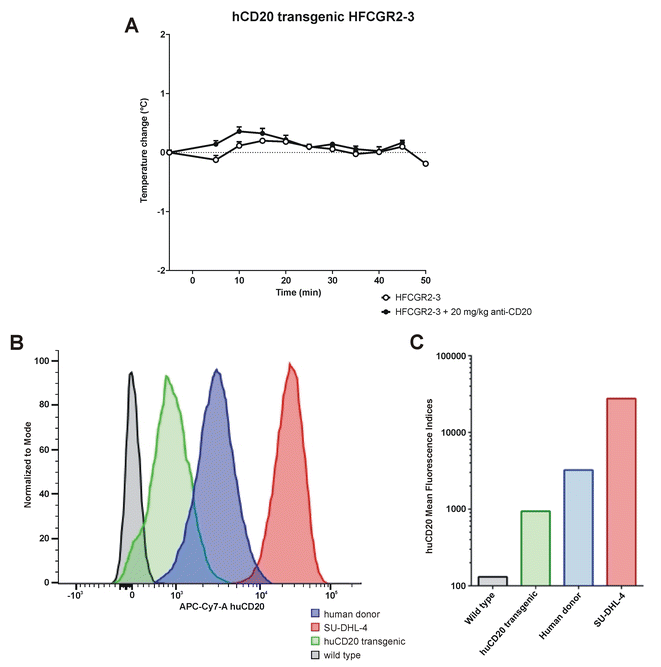

Supplement: Supplementary file 11 — (PNG 272 kb) [file 11095_2018_2448_Fig11_ESM.png]

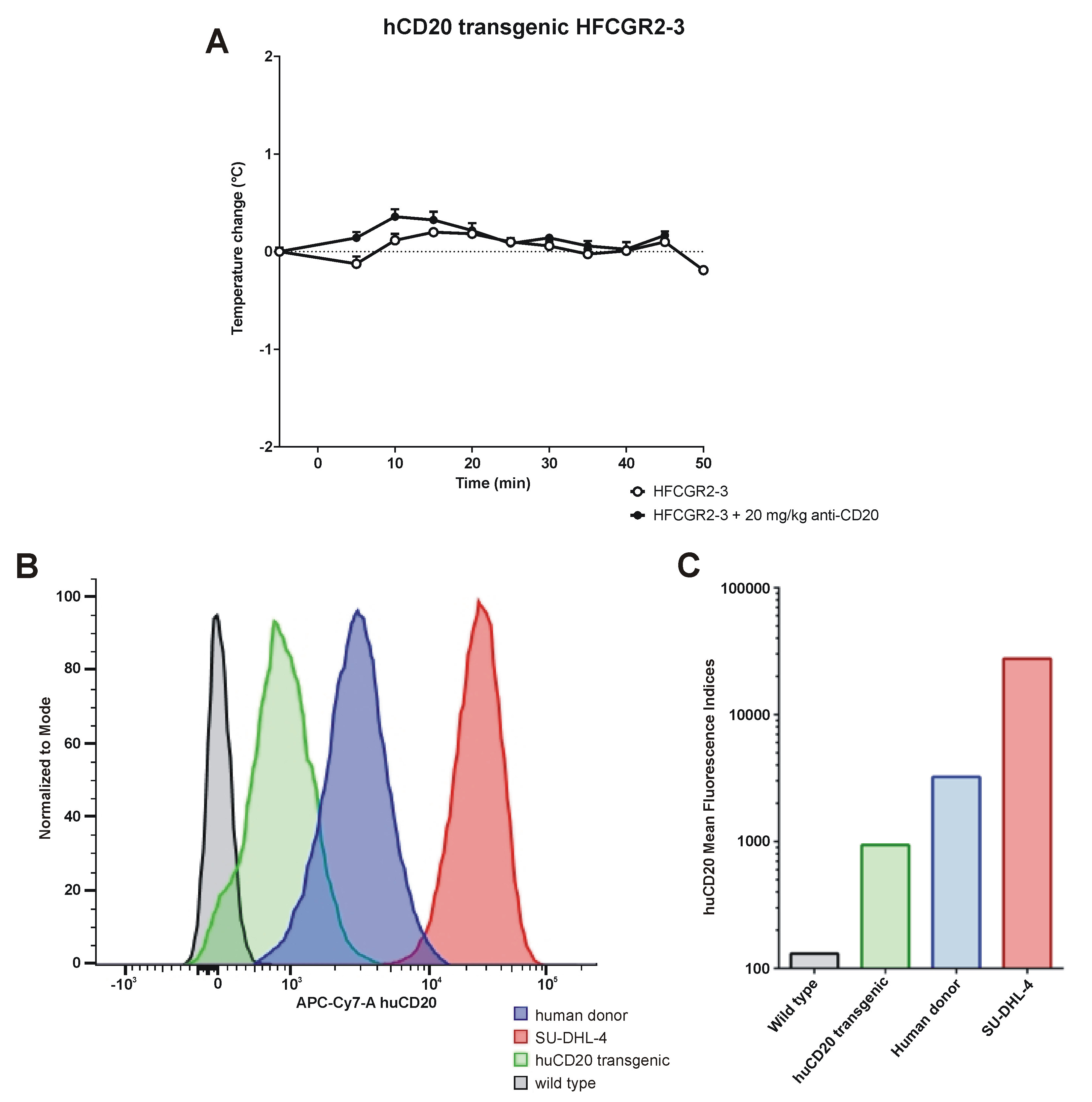

Supplement: Supplementary file 12 — High resolution image (TIF 835 kb) [file 11095_2018_2448_MOESM6_ESM.tif]
