# Supplementary material for: First Infusion Reactions are Mediated by FcγRIIIb and Neutrophils
Source: Pharm Res. 2018 Jun 27;35(9):169. doi: 10.1007/s11095-018-2448-8 (PMC6021477; doi:10.1007/s11095-018-2448-8)
Supplement: Supplementary file 13 — (DOCX 67 kb) [file 11095_2018_2448_MOESM7_ESM.docx]

**First infusion reactions are mediated by FcγRIIIb and neutrophils**

Weber et al.

**SUPPLEMENTARY MATERIAL**

**MATERIALS AND METHODS**

**Generation of HFCGR2-3 and HFCGE3B humanized mice**

Recombination-mediated genomic replacement (RMGR) (1) was used to replace a 48 kb genomic DNA fragment encompassing mouse genes *Fcgr3* and *Fcgr4 (*from 1:171015025 to 1:171062982 of mouse Chr1 in mm10 GRCh38) with a 146 kb fragment of human genomic DNA containing the human genes *FCGR2A*, *FCGR2C*, *FCGR3A* and *FCGR3B (*from 1:161494582 to 1:161640325 of human Chr1_q23.3, in GRCh38). In the BAC vector used for targeting (Fig. 1A, upper line), the human genomic region is flanked by heterotypic LoxP (red triangle) and Lox511 (blue triangle) sites to allow site-specific, cre-mediated recombination with the mouse locus in chromosome 1 which is similarly flanked with LoxP and Lox511 sequence elements (Fig. 1A, middle line). The directed recombination results in the juxtaposition of a promoter-less (silent) Neomycin resistance gene (Neo, within a white arrow) adjacent to the LoxP element inserted into the mouse chromosome with a PGK promoter element inserted beside the LoxP element within the bacterial artificial chromosome (BAC) targeting vector (PGK within a white arrow). The activated PGK-Neo gene permits selection of correctly recombined ES clones. The resulting humanized locus in mouse chromosome 1 lacks the mouse *Fcgr3* and *Fcg4* genes and bears the human *FCGR2A*, *FCGR2C*, *FCGR3A* and *FCGR3B* genes (including all *cis*-acting regulatory elements) as shown in the lower line of Fig. 1A. ES cells bearing the shown genomic replacement were used to generate mutant HFCGR2-3 mice.

The construction of the BAC clone #4 used for RMGR targeting of the murine *FcgR* gene locus is described in Fig. S1 and Fig. S2. Gene targeting vectors for insertion of LoxP and Lox511 elements flanking *Fcgr3* and *Fcgr4,* respectively, were constructed by conventional cloning using pBluescript II SK(+), carrying a diphtheria toxin-A (*DT-A*) expression cassette inserted into the unique *Not*I site, as the backbone. The structure of LoxP and Lox511 targeting vectors is described in detail in Fig. S1 and Fig. S2. The mouse sequences flanking the Lox elements were amplified by PCR from C57/BL6 genomic DNA. Targeting vectors were linearized at a unique restriction site, precipitated with ethanol and solved in PBS prior to electroporation into C57BL/6 ES cells.

The gene targeting construct used to remove the first three exons of the mouse *Fcgr4* gene via directed recombination of a 13 kb fragment of human DNA encompassing 5 kb promoter region, followed by 8 kb bearing the entire human *FCGR3B* gene (from 1:161623196 to 1:161636203 of human Chr1_q23.3 in GRCh38) is depicted in Fig. 1B, upper line. Downstream of the human *FCGR3B* gene a neomycin selection cassette under control of the TK promoter flanked by lox sites is located. The entire fragment of human DNA and Neo selection cassette (Neo marked arrow, flanked by LoxP elements as red triangles) is flanked left by a 3.4 kb arm of homologous mouse DNA (LHA) and right by a 1.8 kb arm of homologous DNA (RHA) to allow directed genomic recombination within the homologous region of mouse chromosome 1 (Fig. 1B, middle line). This targeted recombination causes a deletion of 2.757 kb of mouse sequences from 1:171018254 to :171021011 of Chr 1 in mm10 GRCh38 and replacement of the indicated human DNA fragment. Upon (1.) targeted recombination, Neo resistant ES cells bearing correctly recombined human sequences were treated with (2.) Cre recombinase to delete the Neo gene used for selection (Fig. 1B, lower line). ES cells bearing the correctly excised Neo gene and targeted human *FCGR3B* were used to generate HFCGR3B mutant mice.

**ES cell gene targeting.**

C57/BL6 ES cells (Eurogentech) were cultivated on γ-irradiated murine embryonic fibroblasts (MEFs) in DMEM containing 15% FCS and 20 × 10^6^/l units LIF. For the introduction of foreign DNA, ES cells were resuspended in PBS and electroporated in 0.4 cm cuvettes using a Bio-Rad Gene Pulser instrument set to 0.28 kV/500 μF. For the integration of the LoxP site, 30 μg of linearized targeting vector were electroporated into 30 × 10^6^ ES cells. 48 h after electroporation, positive selection was started using 1 μg/mL puromycin, and resistant colonies were picked into 48-well plates after one week. Targeted clones (tm1 clones) were identified by PCR using specific primers: primer 128 (Table SI), located outside of the homology arm, and primer Neo 1 (Table SI) located inside the drug resistance cassette (PCR conditions: 30 cycles of 10 seconds at 95°C; 1 minute at 64°C and 5 min at 68°C). The identity of the PCR products was confirmed by sequencing. The puromycin resistance cassette was subsequently removed by Cre recombination: 5 × 10^6^ ES cells of tm1 clone IIIB6 were electroporated with 5 μg of the Cre expression vector pMC-Cre, and colonies were picked after one week and screened by PCR (tm1.1 clones) using primers Neo 2 and 107 (Table SI) with the same PCR conditions as above (see amplified PCR products in Fig. S1C). For the integration of the lox511 site, 30 μg of linearized targeting vector were electroporated into 30 × 10^6^ ES cells of tm1.1 clone VF5, followed by puromycin selection, clone picking and identification of targeted clones (tm2 clones) was done by PCR with two primer combinations (primers 148 + Puro 1 and primers Puro 2 + 146, see Table SI; same PCR conditions as above) specific for upstream and downstream targeted regions, respectively (see Fig. S1D, for PCR amplification products).

**Identification of in cis double targeted clones/knock out of *Fcgr3* and *Fcgr4*.**

Six double-targeted ES cell clones (tm2 clones) were analyzed for cis/trans targeting events. For this purpose, a “deleter” construct composed of the PGK promoter flanked by the two LoxP and Lox511 elements in a pBluescript II SK(+) vector was used (Fig. S1E). This construct was co-transfected with the Cre expression vector pMC-Cre into the six selected tm2 clones followed by Neo selection. Correct targeted recombination via Lox elements will generate mutant clones where the 54 kb region containing the *Fcgr3* and *Fcgr4* genes is deleted and the promoter-less Neo gene becomes activated by the insertion of the PGK promoter near the LoxP element of the “deleter” construct (Fig. S1E). We reasoned that recombination of the LoxP/Lox511 elements of the “deleter” construct with the corresponding LoxP/Lox511 in the flanked *FcgR* locus will occur at a much higher frequency in tm2 ES clones having the two Lox elements targeted in cis (in the same chromosome) than in those with the two Lox elements in trans (in different chromosome homologs). 30 h after electroporation, individual neo-resistant colonies from each of the six tm2 clones were picked and expanded for further analysis. Clones carrying the deletion of the *Fcgr3* and *Fcgr4* locus (tm3 clones) were identified by PCR using primers located further outside of the homology sequences used for the integration of LoxP and Lox511 (primers 128 and 146 in Fig. S1E). The result of this screening is summarized in Table SII. The inlet in Fig. S1E, shows an agarose gel demonstrating that the expected DNA fragment of 8.5 kb is amplified from DNA of a tm3 clone (tm), but not from C57BL6 DNA (WT) using primers 128 and 146 reader system (primer sequences in Table SI; PCR amplification conditions: 30 cycles of 10 seconds at 95°C; 1 min at 64; 8 min at 68°C).

**Body temperature telemetry**

Body temperature was done using small battery free telemetric units (IPTT-300) from BMDS with the DAS 7007-S reader system. In brief, chips were implanted and after a recovery period mice were ready to be used in experiments. Temperature measurements were taken at 5 min intervals and were performed in triplicate. Results are displayed as the change in temperature from baseline (measured prior to the experiment).

**Antibodies and reagents**

PerkinElmer inflammation probe was purchased from PerkinElmer Inc. (Waltham, Massachusetts, USA). The LEAF-purified CD11b antibody (clone M1/70) was purchased from Biolegend (San Diego, California, USA). Ly6G neutrophil depleting antibody (clone NIMP-R14) (2) was a gift from Stefan Martin at Freiburg University Medical Center. The antibodies αTfR and αTfR-LALAPG were generated in house by the team of Jens Niewoehner. Rituximab was provided by Christian Klein from Roche RICZ, Zurich. Xolair and Synagis were purchased in pharmacies. The R&D mouse cytokine array panel A was used for cytokine analysis from serum samples. Human lymphoma cell line SU-DHL-4 was obtained from the Roche Cell Bank.

**Flow cytometry**

Fresh whole blood was used for flow cytometry analysis. Red blood cells were lysed using ACK lysis buffer from Lonza. Usually, 50 µL of fresh whole blood was incubated with 3 mL of ACK lysis buffer for 15 min. The following antibodies were used for staining: CD45 (clone 30-F11, BD Biosciences), Gr-1 (clone RB6-8C5, BD Biosciences), F4/80 (clone BM8, BD Biosciences, Nk1.1 (clone PK136, BD Biosciences), mCD16/32 (clone 93, Biolegend), huCD16 (clone 3G8, BD Biosciences), huCD32a/b (clone 3D3, BD Biosciences) , mFcgr4 (clone 9E9, BD Biolegend), B220 (clone RA3-6B2, Biolegend). Cells were incubated for 20 min in the dark at 4°C in a 1:200 dilution of the antibody in FACS buffer (2% FCS in PBS). After staining cells were washed twice in FACS buffer. Data acquisition was performed on a BD FACS Canto I flow cytometer.

**Cytokine determination**

Cytokines were measured using the mouse cytokine antibody array kit panel A from R&D, a 40-plex mouse cytokine analysis tool. The assay was performed according to the manufacturer’s protocol, using 200 µL of pooled serum per group. The kit is semi-quantitative and the data are displayed as % of the assay internal positive control (Table SIII). The results are expressed as the increment value from the control animals in each case. Out of the 40 different cytokines analyzed only KC and MIP-2 displayed a consistent increase as result of the induced FIR, while the other cytokines remained unchanged or revealed an inconsistent change in the different treatments (see Table SIII).Thus, only KC and MIP-2 were considered relevant and are disclosed in the experimental results shown in Figures 2-4. These cytokines are the murine analogues of IL-8 and signal through CXCR2. KC and MIP-2 are strongly associated with neutrophil activation.

**ROS imaging**

Whole body ROS imaging was performed on the PerkinElmer IVIS Spectrum CT. ROS measurements were done using the PerkinElmer inflammation probe. In brief, mice were injected intraperitoneally 10 min prior to their intended time-point of measurement with 170 µL of the PerkinElmer inflammation probe. Test substances were applied directly via i.v. infusion before the imaging. The measurement was performed with an exposure time of 5 min, an aperture of F1 and a medium binning factor of 8. For image analysis the PerkinElmer Living Image Software was used.

**REFERENCES**

1. Wallace H, Marques-Kranc F, Richardson M, Luna-Crespo F, Sharpe J, Hugues J, Wood W, Higgs D, Smith A. Manipulating the mouse genome to engineer precise functional syntenic replacements with human sequence. Cell. 2007;128:197–209.

2. Lopez AF, Strath M, Sanderson, CJ. Differentiation antigens on mouse eosinophils and neutrophils identified by monoclonal antibodies. Br J Haematol. 1984;57:489–94.

**Table SI.** List of primers used.

| **Primer** | **Sequence (5´–3´)** |
| --- | --- |
| 128 | GTTGTCGGGTTAGCCAAGTCC |
| 148 | AGCTGTTGGTAGTAGTAGCTGC |
| 107 | CATGCGGCCGCGTGGTGGAAGTAGCCATTGGC |
| 146 | AAGAGTTGAGCCTCACCTGGG |
| 130 | CCAGCAGAACAGTAACCCCTC |
| 217 | CCACTCAGCAAGCTGAGAGTA |
| 252 | TGCCCTCTAGGGTAGAATCCG |
| 219 | CCACTCAGCAAGCTGAGAGTG |
| 178 | GGGGCAAGCATCCTGGGATG |
| 253 | GCAGGGAGCTCTGGTAGCAG |
| 180 | AATCTCATCCCCAGGGTCTTG |
| 254 | CCGGGATCCCTGGCAGCTG |
| Neo 1 | GCGCATCGCCTTCTATCGCC |
| Neo 2 | GGCGATAGAAGGCGATGCGC |
| Puro 1 | GTCACCGAGCTGCAAGAACTC |
| Puro 2 | GAGTTCTTGCAGCTCGGTGAC |

**Table SII.** Analysis of ES clones for *cis* integration of LoxP and Lox511 elements.

| **Clones** | **Neo resistant** | **PCR positive** |
| --- | --- | --- |
| 1 | 76 | 0 |
| 2 | 372 | 50 |
| 3 | 4000 | 1000 |
| 4 | 3200 | 500 |
| 5 | 112 | 0 |
| 6 | 2800 | 250 |

Identification of in-*cis* targeted ES cell clones. Cre-mediated deletion of the murine Fcgr locus is significantly more efficient when LoxP and Lox511 are located in *cis* (on the same chromosome) rather than in *trans*, allowing unambiguous identification of in-*cis* targeted ES cell clones. The targeted mutation was identified via PCR using primers located in the murine genome outside of the Fcgr locus and outside of the homology arms used for introduction of LoxP and Lox511, respectively (primers 128 and 146, Table SI). Positions of PCR primers size of the expected PCR fragment are indicated in the genomic map of Fig. S2A and S2B.

**Table SIII. Summary of observed cytokine pattern.**

| **Cytokine** | **HFCGR2-3 - WT** | **HFCGR2-3 - HFCGR2-3 dPMN** | **HFCGR2-3 -  WT dPMN** | **FCGR3B - WT** | **HFCGR2-3 -  WT2** |
| --- | --- | --- | --- | --- | --- |
| **KC** | 58.4 | 38.3 | 37.7 | 44.5 | 54.0 |
| **MIP-2** | 49.7 | 65.5 | 66.1 | 24.1 | 19.0 |
| **SDF-1** | 34.8 | 11.3 | 4.0 | 8.9 | 9.5 |
| **JE** | 4.4 | 4.1 | 17.1 | 3.3 | 7.3 |
| **TNF-a** | -0.5 | 19.9 | 16.5 | 2.7 | 3.7 |
| **IL-27** | 5.9 | 0.3 | 0.1 | 1.6 | 2.7 |
| **IL-5** | 6.0 | 2.6 | 4.1 | 0.5 | 1.8 |
| **RANTES** | 9.4 | 7.0 | 5.4 | 0.5 | 1.6 |
| **MCP-5** | 4.7 | 12.3 | 14.9 | 1.4 | 1.4 |
| **s-ICAM-1** | -0.3 | 2.8 | 3.7 | -0.7 | 0.5 |
| **IL-6** | 7.7 | 3.9 | 5.3 | 0.7 | 0.1 |
| **IL-3** | 7.7 | 0.7 | 1.8 | -0.8 | 0.0 |
| **MIP-1a** | 2.7 | 9.0 | 9.0 | 1.1 | -0.1 |
| **MIG** | 3.6 | -9.9 | 11.7 | 0.9 | -0.1 |
| **IL-17** | 5.4 | 2.6 | 2.8 | 1.3 | -0.1 |
| **IL-10** | 4.4 | 4.9 | 6.2 | 2.3 | -0.2 |
| **MIP1b** | -0.2 | 9.1 | 8.6 | -1.0 | -0.3 |
| **IL-12p70** | 4.1 | 0.7 | 0.7 | 0.1 | -0.5 |
| **IFN-g** | -3.1 | 3.3 | 3.3 | 0.0 | -0.6 |
| **TARC** | 7.6 | 2.2 | 2.8 | -1.2 | -0.6 |
| **I-TAC** | 8.1 | 0.9 | 2.3 | -1.1 | -0.6 |
| **M-CSF** | 10.4 | 12.9 | 9.7 | -3.9 | -0.6 |
| **IL-1b** | 2.7 | 3.7 | 4.1 | 4.4 | -0.6 |
| **IL-7** | 5.5 | 7.2 | 8.1 | -1.1 | -0.7 |
| **IL-23** | 4.7 | 0.9 | 1.0 | 0.3 | -0.7 |
| **IL-2** | 5.8 | 0.3 | 0.9 | 0.4 | -0.8 |
| **IL-4** | 8.5 | 1.8 | 3.3 | -0.9 | -0.9 |
| **G-CSF** | 51.4 | 5.3 | 25.1 | 1.5 | -1.3 |
| **I-309** | 2.8 | 3.1 | 4.7 | -1.3 | -1.4 |
| **Eotaxin** | 3.7 | -1.3 | 1.9 | -0.7 | -1.6 |
| **IL-1a** | 0.3 | 32.0 | 25.0 | 4.9 | -1.7 |
| **GM-CSF** | 0.3 | 2.7 | 3.2 | -2.0 | -1.8 |
| **TREM-1** | 5.5 | 8.0 | 8.6 | -4.5 | -2.4 |
| **IL-13** | 3.3 | 2.1 | 5.6 | -1.3 | -3.4 |
| **IL-1Ra** | -13.3 | 1.3 | 13.4 | 10.9 | -3.6 |
| **IP-10** | -2.7 | 6.0 | 2.5 | -6.1 | -4.0 |
| **IL-16** | 9.7 | 21.9 | 23.1 | 5.9 | -8.7 |
| **C5a** | 2.3 | 7.9 | 10.1 | -0.7 | -9.1 |
| **TIMP-1** | 1.7 | 5.9 | -2.4 | -20.8 | -16.7 |
| **BLC** | 6.3 | 2.3 | 3.0 | -47.6 | -37.4 |

The table displays the cytokine values measured in the experiments of Figures 2B, 3C and 4B, as described in M&M, and after subtraction of the value of the corresponding (untreated or wild type) control group in each experiment, indicated in the table heading as “HFCGR2-3 – WT”, “HFCGR2-3 – HFCGR2-3dPMN”, etc. The two HFCGR2-3 – WT values in the table reflect the two measurements in Figures 2B and 3B, respectively. The red and blue shading are arbitrary colors given to indicate increase or decrease, respectively and white indicates no/low changes. dPMN, neutrophil-depleted; WT, wild type.

**LEGENDS TO FIGURES**

**Figure S1. Targeted insertion of LoxP and Lox511 elements flanking mouse *Fcgr3* and *Fcgr4*.** **A,** A linearized vector, containing a promotor less neomycin resistance gene (*neo*, within the thick open arrow) and a puromycin resistance cassette (*Tk-puro,* within an open rectangle) flanked by LoxP elements (red triangles) for positive selection, and two mouse DNA homology arms (thick gray bars, 3.2 and 1.9 kb long, respectively), was integrated into the murine genome upstream of exon 1 (gray box, labelled E1) of *Fcgr3* by means of gene targeting. In a second step, the puromycin resistance cassette was removed by Cre-mediated recombination leaving behind the promotor-less (silent) neomycin resistance gene and a single LoxP site. **B,** A linearized vector, containing a puromycin resistance cassette (*Tk-puro*) for positive selection, a thymidine kinase expression cassette (*Tk-tk*) for negative selection, and a single Lox511 element (blue triangle), flanked by homology arms (thick gray bars, 2 and 2.5 kb long, respectively), was integrated into the murine genome upstream of exon 1 (gray box, labelled E1) of *Fcgr4* by means of gene targeting. **C,** PCR analysis of an ES cell clone obtained by gene targeting as described in A. The agarose gel shows the DNA fragments specifically PCR-amplified from the targeted mutated gene locus (Tm) as opposed to the unmutated locus (WT), with indication of the Tm fragment size before (-Cre: 4 kb) and after Cre recombination (+Cre: 2.8 kb). PCR reactions were done using specific primer combinations at the 5’- and 3’-ends: primers 128 and 107 are located outside of the homology arms, primers Neo1 and Neo2 are located within the *neo* resistance gene in opposite orientation. Positions of PCR primers and sizes of expected PCR fragments are indicated in A. **D,** PCR analysis of an ES cell clone obtained by gene targeting as described in B. The agarose gel shows the DNA fragments specifically PCR-amplified from the targeted mutated gene locus (Tm) as opposed to the unmutated locus (WT). The targeted mutation (Tm) was identified by PCR using specific primer combinations at the 5’- and 3’-ends: primers 148 and 146 are located outside of the homology arms, primers Puro1 and Puro2 are located within the *Tk-puro* resistance cassette, in opposite orientation. Positions of PCR primers and sizes of the expected PCR fragments are indicated in A and B. Mouse homology arms were obtained by PCR amplification using specific primers (see Table SI). Sizes of marker fragments (M) in the agarose gels are indicated. Diagrams are not drawn to scale. **E.** Deletion of *Fcgr3* and *Fcgr4* and identification of in *cis* targeted ES cell clones. The murine Fcgr locus – flanked by heterotypic LoxP and Lox511 sites – was removed by cre-mediated cassette exchange using a “deleter” construct (upper line). Recombination at LoxP places a PGK promoter (located on the “deleter” construct) in front of the promoter-less (silent) neomycin resistance gene (*Neo*, located on the murine chromosome, see also Fig. S1A), allowing positive selection. Correct recombination at Lox511 leads to the removal of the chromosomal thymidine kinase expression cassette (*Tk-tk*), rendering ES cells FIAU resistant. Cre-mediated deletion of the murine Fcgr locus is significantly more efficient when LoxP and Lox511 are located in *cis* (on the same chromosome) rather than in *trans* (see Table SII and Materials and Methods section), allowing unambiguous identification of in *cis* targeted ES cell clones. The resulting targeted mutation can be identified via PCR using primers 128 and 146 as an 8,5 kb DNA amplicon and is absent in non-deleted ES clones (lower line and Tm in the inlet). Size of marker fragments (M) in the agarose gels is indicated. Diagrams are not drawn to scale.

**Figure S2. Construction of a bacterial artificial chromosome (BAC) containing human *FCGR2A*, *FCGR3A*, *FCGR2C*, and *FCGR3B***. The BAC clone #4 used in Fig. 1 for recombination-mediated genomic replacement (RMGR) was assembled from two overlapping BACs, RP11-25K21 (harboring *FCGR3A*, *FCGR2C*, *FCGR3B*, and *FCGR2B*) and RP11-5K23 (harboring *FCGR2A* and *FCGR3A*). BAC vector backbone elements are displayed translucent, and the *EcoR*I sites that had been used to clone the BAC insert during library construction are indicated. **A,** *FCGR2B*, as well as a *SacBII* cassette and a LoxP site located on the BAC vector backbone (translucent) were deleted from BAC #1 (RP11-25K21, see linear scheme in C) by recombineering using a β-lactamase cassette (*Bla*) and a Lox511 site flanked by short homology arms (50 bp, thick white or translucent bars). The resulting BAC #2 carries a Lox511 site 8.9 kb upstream of *FCGR3B*(see linear scheme in C). **B,** A 46 kb DNA fragment, subcloned from BAC RP11-5K23 and containing a floxed neomycin resistance cassette (*Neo*), a *PGK* promoter (in reverse orientation), and *FCGR2A*, flanked by homology arms, was integrated into BAC #2 by recombineering. One homology arm of 0.6 kb targeted the chloramphenicol acetyltransferase gene (*CAT*) located on the BAC vector backbone (translucent), thereby also eliminating a Lox511 site located on the vector backbone. The other homology arm, which is part of the overlap between the two original BAC clones, targeted the first 2.6 kb of the BAC insert (thick white bar). The resulting BAC #3 (see also linear schem in C) contains a floxed neomycin resistance cassette and a *PGK* promoter roughly 11 kb upstream of *FCGR2A*, while the intergenic region between *FCGR2A* and *FCGR3A* is restored. In the last step, the neomycin resistance cassette was removed from BAC #3 by Cre-mediated recombination, leaving behind the *PGK* promoter next to a single LoxP site and yielding BAC #4, the BAC clone used in RMGR (see linear scheme in C). **C,** Linear schematic representation of the human *FCGR* locus (top) and of BAC clones #1 to #4, drawn to scale. Positions of *Ksp*I restriction sites, as well as expected restriction fragment sizes are indicated for the four different BAC clones. **D,** *Ksp*I restriction digest of the four BAC clones analyzed on a 0.4 % agarose gel. Lane M1: Roche marker XV; lane 1: BAC #1; lane 2: BAC #2; lane 3: BAC #3; lane 4: BAC #4; lane M2: Roche marker IV. **E**, PCR amplification of human *FCGR* genes from knock-in animals: human *FCGR2A*, *FCGR3A*, *FCGR2C* and *FCGR3B* were amplified by PCR from genomic DNA of heterozygous knock-in animals using specific primer combinations: Primers 130 + 217 were used for amplification of human *FCGR2A*; primers 252 + 219 for amplification of human *FCGR2*C gene; primers 178 + 253 for amplifying human *FCGR3A* and primers 180 + 254 for amplifying human *FCGR3B* (see primer sequences in Table SI; PCR conditions: 30 cycles of 15 seconds at 94°C; 30 seconds at 64°C and 15 minutes at 68°C). PCR fragments of 12.9 kb, 9.6 kb, 18.5 kb and 9.6 kb are expected for *FCGR2A*, *FCGR3A*, *FCGR2C* and *FCGR3B*, respectively. **F**, Digestion of the two identical 9.6 kb *FCGR3A* and *FCGR3B* PCR fragments with restriction enzyme *Sal*I is used to identify these two genes. The *FCGR3A* fragment is cut by *Sal*I, giving rise to 8.0 kb and 1.6 kb fragments (arrow), while the *FCGR3B* fragment does not contain a *Sal*I site and remains as 9.6 kb band. Diagrams in A and B are not drawn to scale.

**Figure S3. HFCGR2-3 and wild type mice analysis for expression of human CD16 by flow cytometry**. **A,** The expression of human CD16 in Gr-1 positive (neutrophils), F4/80 positive (monocytes) and Nk1.1 positive (NK) cells is depicted. HFCGR 2-3 and wild type mice were analyzed for the expression of human CD32 by flow cytometry. **B,** The expression of human CD32 in Gr-1 positive, F4/80 positive and Nk1.1 positive cells is depicted.

**Figure S4. Expression pattern of human FcγR3b in HFCGR3B mice.** **A**, The expression of human CD16 in Gr-1 positive cells (neutrophils), F4/80 positive cells (monocytes) and Nk1.1 positive cells (NK cells) is depicted. **B,** Expression of mouse CD16/32 and mouse FcγR4 in neutrophils of HFCGR2-3, HFCGR3B and wild type mice. **C,** Expression of mouse FcγR4 in HFCGR2-3, HFCGR3B and wild type mice. Expression in Gr-1 positive, F4/80 positive and Nk1.1 positive cells are depicted.

**Figure S5. A. Comparison of i.v. versus s.c. antibody injection. A,** Change of body temperature of HFCGR2-3 mice injected with 20 mg/kg HamTfR either i.v. or s.c. **B,** Antibodies lacking resident targets fail to induce FIR. Change of body temperature of HFCGR2-3 mice injected with 250 mg/kg Xolair or Synagis. **C,** Interaction of infused antibodies with *FcgR*s is crucial to elicit FIR. Change of body temperature of HFCGR2-3 mice injected with 20 mg/kg HamTfR-LALAPG or with solvent buffer.

**Figure S6**. **A,** No signs of FIR upon infusion of Rituximab in hCD20 transgenic HFCGR2-3 mice. Change of body temperature of HFCGR2-3 mice crossed with human CD20-transgenic mice after infusion of 20 mg/kg anti-CD20 mAb Rituximab. **B,** Expression levels of human CD20 in the transgenic mice, in human peripheral blood B cells from a healthy donor and in SU-DHL-4 lymphoma cells. **C,** Mean fluorescence indices (MFI) corresponding to the expressed levels of human CD20 detected in.
